# The role of antibody responses against glycans in bioprosthetic heart valve calcification and deterioration

**DOI:** 10.1038/s41591-022-01682-w

**Published:** 2022-02-17

**Authors:** Thomas Senage, Anu Paul, Thierry Le Tourneau, Imen Fellah-Hebia, Marta Vadori, Salam Bashir, Manuel Galiñanes, Tomaso Bottio, Gino Gerosa, Arturo Evangelista, Luigi P. Badano, Alberto Nassi, Cristina Costa, Galli Cesare, Rizwan A. Manji, Caroline Cueff de Monchy, Nicolas Piriou, Romain Capoulade, Jean-Michel Serfaty, Guillaume Guimbretière, Etienne Dantan, Alejandro Ruiz-Majoral, Guénola Coste du Fou, Shani Leviatan Ben-Arye, Liana Govani, Sharon Yehuda, Shirley Bachar Abramovitch, Ron Amon, Eliran Moshe Reuven, Yafit Atiya-Nasagi, Hai Yu, Laura Iop, Kelly Casós, Sebastián G. Kuguel, Arnau Blasco-Lucas, Eduard Permanyer, Fabrizio Sbraga, Roger Llatjós, Gabriel Moreno-Gonzalez, Melchor Sánchez-Martínez, Michael E. Breimer, Jan Holgersson, Susann Teneberg, Marta Pascual-Gilabert, Alfons Nonell-Canals, Yasuhiro Takeuchi, Xi Chen, Rafael Mañez, Jean-Christian Roussel, Jean-Paul Soulillou, Emanuele Cozzi, Vered Padler-Karavani

**Affiliations:** 1 Institut du Thorax, Institut National de la Santé et de la Recherche Médicale UMR1087, University Hospital, Nantes, France; 2 Institut National de la Santé et de la Recherche Médicale UMR 1246-SPHERE, Nantes University, Tours University, Nantes, France; 3 Department of Cell Research and Immunology, Shmunis School of Biomedicine and Cancer Research, George S. Wise Faculty of Life Sciences, Tel Aviv University, Tel Aviv, Israel; 4 Consortium for Research in Organ Transplantation, Ospedale Giustinianeo, Padova, Italy; 5 Department of Cardiac Surgery and Reparative Therapy of the Heart, Vall d’Hebron Research Institute, University Hospital Vall d’Hebron, Universitat Autònoma de Barcelona, Barcelona, Spain; 6 Cardiovascular Regenerative Medicine Group, Department of Cardiac, Thoracic and Vascular Surgery, University of Padova, Padova, Italy; 7 Department of Cardiac, Vascular and Thoracic Sciences and Public Health University of Padova, L.I.F.E.L.A.B. Program Veneto Region, Padova, Italy; 8 Department of Cardiology, Vall d’Hebron Research Institut, Hospital Vall d’Hebron, Barcelona, Spain; 9 Department of Medicine and Surgery, University of Milano-Bicocca, Milan, Italy; 10 Department of Cardiology, Neural and Metabolic Sciences, Istituto Auxologico Italiano, Istituto di Ricovero e Cura a Carattere Scientifico, San Luca Hospital, Milan, Italy; 11 Transplantation Immunology Unit, Padova University Hospital, Padova, Italy; 12 Infectious Diseases and Transplantation Division, Institut d’Investigació Biomèdica de Bellvitge, L’Hospitalet de Llobregat, Barcelona, Spain; 13 Avantea, Cremona, Italy; 14 Department of Surgery, Max Rady College of Medicine, University of Manitoba Cardiac Sciences Program, St Boniface Hospital, Winnipeg, Manitoba Canada; 15 Department of Cardiology, Bellvitge University Hospital, L’Hospitalet de Llobregat, Barcelona, Spain; 16 Department of Chemistry, University of California, Davis, Davis, CA USA; 17 Cardiac Surgery Department, Bellvitge University Hospital, L’Hospitalet de Llobregat, Barcelona, Spain; 18 Pathology Department, Bellvitge University Hospital, L’Hospitalet de Llobregat, Barcelona, Spain; 19 Intensive Care Department, Bellvitge University Hospital, L’Hospitalet de Llobregat, Barcelona, Spain; 20 Mind the Byte, Barcelona, Spain; 21 Department of Surgery, Institute of Clinical Sciences, Sahlgrenska Academy at the University of Gothenburg, Gothenburg, Sweden; 22 Institute of Biomedicine, Department of Laboratory Medicine, Sahlgrenska Academy at the University of Gothenburg, Gothenburg, Sweden; 23 Institute of Biomedicine, Department of Medical Biochemistry and Cell Biology, Sahlgrenska Academy at the University of Gothenburg, Gothenburg, Sweden; 24 Institut Universitari de Ciència i Tecnologia (Inkemia Group), Barcelona, Spain; 25 Division of Infection and Immunity, University College London, London, UK; 26 Institut de Transplantation-Urologie-Néphrologie, Institut National de la Santé et de la Recherche Médicale Unité Mixte de Recherche 1064, Centre Hospitalier Universitaire de Nantes, Nantes, France; 27 Present Address: Department of Pathology, Brigham and Women’s Hospital, Harvard Medical School, Boston, MA USA; 28 Present Address: Israel Institute for Biological Research, Ness Ziona, Israel; 29 Present Address: Department of Cardiac Thoracic and Vascular Sciences and Public Health, University of Padova, Padova, Italy; 30 Present Address: Department of Cardiovascular Disease at the Vall d’Hebron Institut Research, University Hospital Vall d’Hebron, Universitat Autònoma de Barcelona, Barcelona, Spain; 31 Present Address: Department of Cardiac Surgery, Quironsalud Teknon Heart Institute, Barcelona, Spain; 32 Present Address: Molomics, Barcelona, Spain; 33 Present Address: DevsHealth, Barcelona, Spain

**Keywords:** Outcomes research, Risk factors

## Abstract

Bioprosthetic heart valves (BHVs) are commonly used to replace severely diseased heart valves but their susceptibility to structural valve degeneration (SVD) limits their use in young patients. We hypothesized that antibodies against immunogenic glycans present on BHVs, particularly antibodies against the xenoantigens galactose-α1,3-galactose (αGal) and *N*-glycolylneuraminic acid (Neu5Gc), could mediate their deterioration through calcification. We established a large longitudinal prospective international cohort of patients (*n* = 1668, 34 ± 43 months of follow-up (0.1–182); 4,998 blood samples) to investigate the hemodynamics and immune responses associated with BHVs up to 15 years after aortic valve replacement. Early signs of SVD appeared in <5% of BHV recipients within 2 years. The levels of both anti-αGal and anti-Neu5Gc IgGs significantly increased one month after BHV implantation. The levels of these IgGs declined thereafter but anti-αGal IgG levels declined significantly faster in control patients compared to BHV recipients. Neu5Gc, anti-Neu5Gc IgG and complement deposition were found in calcified BHVs at much higher levels than in calcified native aortic valves. Moreover, in mice, anti-Neu5Gc antibodies were unable to promote calcium deposition on subcutaneously implanted BHV tissue engineered to lack αGal and Neu5Gc antigens. These results indicate that BHVs manufactured using donor tissues deficient in αGal and Neu5Gc could be less prone to immune-mediated deterioration and have improved durability.

## Abstract (web summary)

In a large cohort of patients who underwent aortic valve replacement, antibody responses to glycans present in bioprosthetic heart valves, notably galactose-α1,3-galactose and *N*-glycolylneuraminic acid, were implicated in valve calcification and deterioration.

## Main

Heart valve disorders are among the most common cardiovascular diseases. Aortic valve stenosis represents >40% of valvular heart diseases affecting 2% of the Western population^1^. Curative valve replacement is the second most frequent cardiac operation after coronary artery bypass grafting^2^. Implantation of animal-derived BHVs has increased, replacing mechanical heart valves^1^. This is due to the aging population, improvement in bioprostheses hemodynamic performance and development of percutaneous transcatheter aortic valve implantation (TAVI)^3^. Unlike mechanical heart valves, BHVs do not require lifelong patient anticoagulation but have limited durability due to SVD occurring approximately 10–12 years after implantation^4^. SVD pathophysiology is mediated by progressive leaflet tissue stiffening and calcification, eliciting valve narrowing and/or leakage (stenosis/regurgitation)^5^. This is suggested to be initiated by passive accumulation of calcium or by an atherosclerotic-like process^5^. Deleterious immune responses against xenogeneic antigens in BHVs could also contribute to calcification and eventual SVD^6^. While investigated in mice^7–9^ and humans^7,10^, direct evidence in patients is still pending. Despite pretreatment of commercial BHVs to reduce immunogenicity against animal-derived proteins, residual xenogenic carbohydrate antigens remain exposed^7,11,12^.

αGal is a carbohydrate antigen expressed in animal-derived tissues and cells; anti-αGal antibodies trigger hyperacute xenotransplant rejection in nonhuman primates^13^. Since all humans have circulating anti-αGal antibodies^14^, an αGal antigen had been suggested as a target of immune-mediated BHV failure^7,11,15^. However, while αGal knockout tissue grafts reduced the immune response against BHVs, they did not eliminate it^15^, prompting search for other immunogenic residues in BHVs^15,16^. Neu5Gc is another carbohydrate xenoantigen^16^ suggested to be a potent non-αGal immunogen since humans also have circulating anti-Neu5Gc antibodies^17,18^. Neu5Gc is a sialic acid covering the tips of glycans on various glycoproteins and glycolipids, attached via α2-3/6/8-linkages^19^. In fact, tissues used for BHV production (bovine and equine pericardia or porcine aortic valve leaflets) contain diverse glycosphingolipids/glycoproteins^20^ with Neu5Gc or αGal^21^, also in porcine heart valves^22^. Bovine pericardia express the highest levels of αGal/Neu5Gc-*N*-glycans^20^. Both αGal and Neu5Gc are immunogenic, cannot be synthesized in humans each due to a specific gene inactivation and are suggested to impact transplant rejection risk^23^ and BHV deterioration^24^.

Translink, a European Union-funded project, assessed the role of these xenoantigens in BHV deterioration. This is a large longitudinal prospective international study with enrollment of patients before treatment through 15 years post-aortic BHV replacement. We investigated BHV calcification, SVD, anti-αGal and anti-Neu5Gc IgGs in patients over time. We also documented these xenoglycans and antibodies in calcified valves explanted from patients. Finally, we investigated the involvement of anti-glycan antibodies at inducing calcification of valve tissues in xenoglycan-deficient mice.

## Results

### Translink cohort

**Fig. 1:**
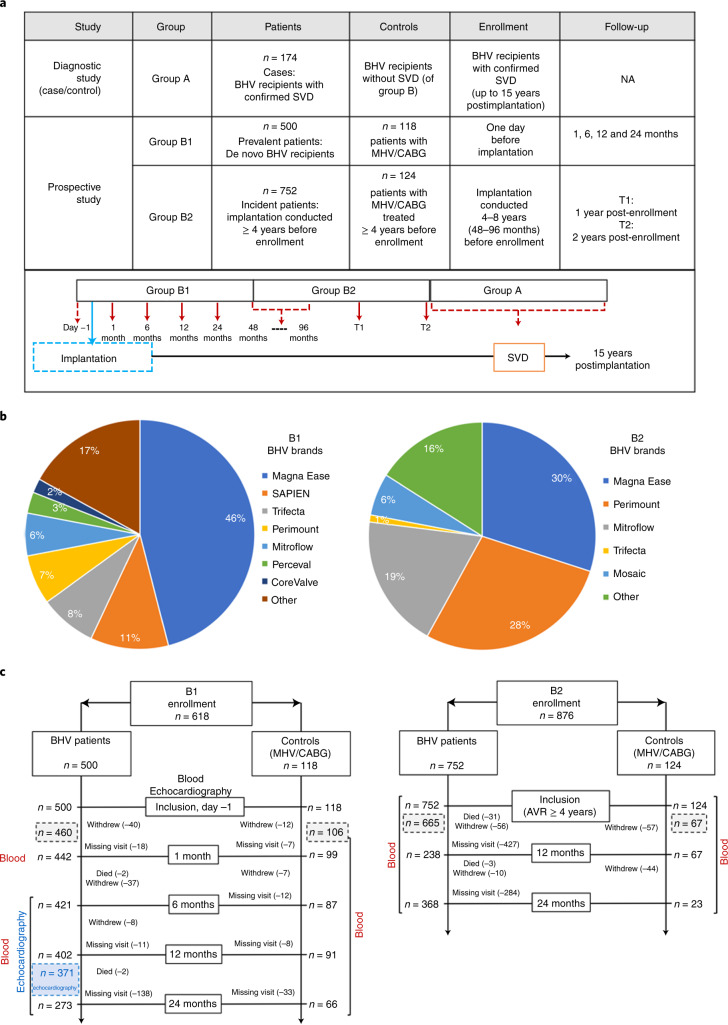
Description of the Translink cohort. **a**, Top: Table describing the patient groups in the Translink study. This included three groups of BHV recipients (groups A, B1 and B2) and control patients who were treated with MHV replacement surgery or CABG. Bottom: Timeline of patients enrollment and follow-up. **b**, Distribution of BHV brands implanted into patients in groups B1 and B2. **c**, Flowcharts describing the two arms (BHV patients and control patients) in groups B1 and B2. The total number of patients who remained in the study after hospital discharge for group B1 or after the inclusion visit for group B2 is shown (gray dashed boxes). The number of BHV recipients who had an echocardiography at the end of the group B1 follow-up are shown (blue dashed box).

The Translink multicenter study was designed to investigate early and long-term humoral immune responses elicited by implanted aortic BHVs up to approximately 15 years postimplantation (Fig. 1a) and their clinical impact. In this diagnostic/prospective clinical study patients were enrolled into four groups: (1) group B1 (*n* = 500) included de novo BHV recipients who had diseased valve replacement either by surgery or TAVI, with serial follow-up at 1, 6, 12 and 24 months after aortic valve replacement (AVR); (2) group B2 (*n* = 752) included patients who had BHV implantation at least 4 years before inclusion, with further follow-up through 24 months; (3) control groups were non-BHV recipients, who went through either implanted mechanical heart valve (MHV) replacement surgery or coronary artery bypass graft (CABG), for both group B1 (*n* = 118: *n* = 57 MHV recipients, *n* = 61 patients with CABG) and group B2 (n = 124: n = 43 MHV-recipients, n = 81 CABG patients); (4) group A (*n* = 174) included BHV recipients diagnosed with SVD (Fig. 1a and Table 1; Supplementary Table 1 describes the inclusion and exclusion criteria). Group B2 patients were enrolled at 4.0–14.5 years after AVR (BHV patients: 6.9 ± 1.7 years; control patients: 7.1 ± 1.7 years).

**Table 1:** Baseline characteristics of the Translink cohort at inclusion

	Group A	Group B1			Group B2		
Characteristics	BHV SVD patients	BHV patients	Controls	*P*	BHV patients	Controls	*P*
	*n* = 174	*n* = 500	*n* = 118		*n* = 752	*n* = 124	
Age (years)	77 ± 10	74 ± 8	64 ± 11	<0.001	78 ± 8	70 ± 11	<0.0001
Male, *n* (%)	92 (52.9)	302 (60.5)	93 (78.8)	<0.001	508 (67.6)	102 (82.3)	0.001
SBP, mmHg	134 ± 21	132 ± 17	131 ± 17	0.93	138 ± 18	NA	
NYHA class 2–4, *n* (%)	145 (84.3)	437 (87.4)	77 (65.3)	<0.001	246 (34.1)	30 (24.4)	0.034
BMI, kg m^−2^	28 ± 5	28.1 ± 4.4	27.6 ± 4.2	0.55	28 ± 4	28 ± 4	0.158
Tobacco, *n* (%)	60 (34.5)	179 (35.8)	65 (55.1)	<0.001	234 (31.2)	31 (25.0)	0.164
Hypertension, *n* (%)	139 (79.9)	401 (80.2)	89 (75.4)	0.25	619 (82.4)	92 (12.9)	0.030
Diabetes, *n* (%)	49 (28.2)	127 (25.4)	29 (24.6)	0.85	196 (26.1)	32 (25.8)	0.945
Dyslipidemia, *n* (%)	NA	18 (15)	46 (16)	0.36	NA	NA	
Creatinine clearance, ml/min/1.73 m^−2^	62 ± 25	74 ± 21	85 ± 23	0.26	67 ± 23	83 ± 26	0.061
CPB time, min	NA	111 ± 59	102 ± 49	0.58	NA	NA	
PPM							
Moderate, *n* (%)	NA	171 (34.2)	NA		NA	NA	
Severe, *n* (%)	NA	15 (3.0)	NA		NA	NA	
Intervention, *n* (%)							
BHV	170 (97.7)	429 (85.8)	–		746 (99.2)	–	
TAVI	4 (2.3)	71 (14.2)	–		6 (0.8)	–	
CABG	–	95 (19.0)	61 (51.7)		193 (25.7)	43 (34.7)	
MHV	–	–	57 (48.3)		–	81 (65.3)	
Center, *n* (%)							
Nantes University Hospital, France	125 (71.8)	185 (37.0)	34 (28.8)		317 (42.6)	12 (9.7)	
Azienda Ospedaliera di Padova, Italy	8 (4.6)	141 (28.2)	25 (21.2)		184 (24.7)	55 (44.4)	
University Hospital Vall D’Hebron, Spain	35 (20.1)	123 (24.6)	41 (34.7)		174 (23.4)	37 (29.8)	
Bellvitge University Hospital, Spain	6 (3.4)	51 (10.2)	18 (15.2)		70 (9.4)	20 (16.1)	
St Boniface Hospital, Winnipeg, Canada	–	–	–		7 (0.9)	–	
				0.02			<0.001

Group A: SVD patients; Group B1: first-time BHV recipient or control MHV or CABG treated patients; Group B2: BHV recipients ≥4 years before inclusion and controls (MHV recipients or patients treated with CABG ≥4 years before inclusion). Comparisons between groups were performed using Student’s *t*-tests or chi-squared tests for continuous or categorical variables, respectively. NA, not applicable; NYHA, New York Heart Association; SBP, systolic blood pressure.

Enrollment in the Translink study and patient follow-up were performed at 4 major European cardiovascular surgery centers in France, Italy, Spain (*n* = 2) and in Canada (Table 1). In each center, BHV recipients received any of the most widely used and commercially available BHVs (Fig. 1b), mainly made of bovine pericardium (approximately 80%; CoreValve/Mosaic/Hancock II of porcine origin). At enrollment, the demographics and physiological characteristics of participants were recorded, revealing several differences between patients and controls (Table 1). Subsequently, the patients enrolled in groups B1 and B2 were followed by echocardiography for hemodynamic, leaflet function and calcification; blood samplings were taken to assess antibody responses against αGal and Neu5Gc (Fig. 1c). The complete Translink cohort included 1,668 patients with a total of 4,998 blood samples examined further.

### Assessment of BHV SVD and calcification

In group B1 (de novo BHV recipients), before the diseased native aortic valve was replaced it was assessed by echocardiography at baseline (*n* = 500; Supplementary Table 2). Then, after valve replacement, the implanted BHV was assessed by echocardiography during each follow-up visit (at months 6 and 12–24; Supplementary Table 2). Conversely, a computed tomography (CT) scan calcium scoring of BHV was recorded once at the end of follow-up in a subgroup of these patients (*n* = 123; Supplementary Table 2). Initially, based on the implanted BHV model and size indexed to the patient’s body surface area, a moderate patient-prosthesis mismatch (PPM) was found in 34.2% of group B1 BHV patients (0.65–0.85 cm^−2^ m^−2^; *n* = 171 out of 500) but severe prosthesis mismatch (<0.65 cm^−2^ m^−2^) was observed in only 3% of patients (*n* = 15 out of 500). At baseline, there were no differences in the echocardiography of the diseased native aortic valve (in patients before surgery with either BHV or MHV; Supplementary Table 2). Peak transprosthetic velocity and the mean gradient of BHV measured at 12–24 months after surgery were slightly higher than those measured at 6 months (*P* = 0.003 and *P* < 0.0001, respectively), while the effective orifice area was slightly smaller (*P* = 0.008; Supplementary Table 2).

Based on echocardiography, early signs of SVD were found in 4.9% (18 out of 371) of assessed patients after 21 ± 5 months. Compared to the remaining 353 patients without early signs of SVD, the measurements in these 18 early-SVD patients showed that peak aortic BHV velocity increased by >50% (from 2.3 ± 0.5 m s^−1^ to 3.4 ± 0.4 m s^−1^, *P* < 0.0001), mean gradient increased by >50% (from 12.5 ± 5.1 mmHg to 28.1 ± 9.0 mmHg, *P* < 0.0001), while the effective orifice area was reduced by >40% (from 1.89 ± 0.53 cm^2^ to 1.03 ± 0.29 cm^2^, *P* < 0.0001). In a subgroup of these patients who were also assessed by CT scan, 8.1% (10 out of 123) had some degree of calcification, with calcium scoring of 8–144 Agatston units (standard unit for calcium scoring by CT scan). Thus, the echocardiography and CT scans of group B1 patients confirmed that some patients developed early SVD during the first two years of follow-up, suggesting that early pathophysiological events in patients could contribute to calcification onset. We next evaluated changes in humoral immune responses, particularly within the first two years postimplantation.

### Assessment of humoral immune responses against BHV

Carbohydrate xenoantigens expressed on BHV source tissues^20–22^ are resistant and unshielded by tissue fixation agents and recognized by human antibodies^7,11,12^. Antibodies against αGal and Neu5Gc xenoantigens were measured in 4,998 blood samples of the Translink cohort (most samples were accessible at inclusion and less so during follow-up; Fig. 1c and Supplementary Table 3).

**Fig. 2:**
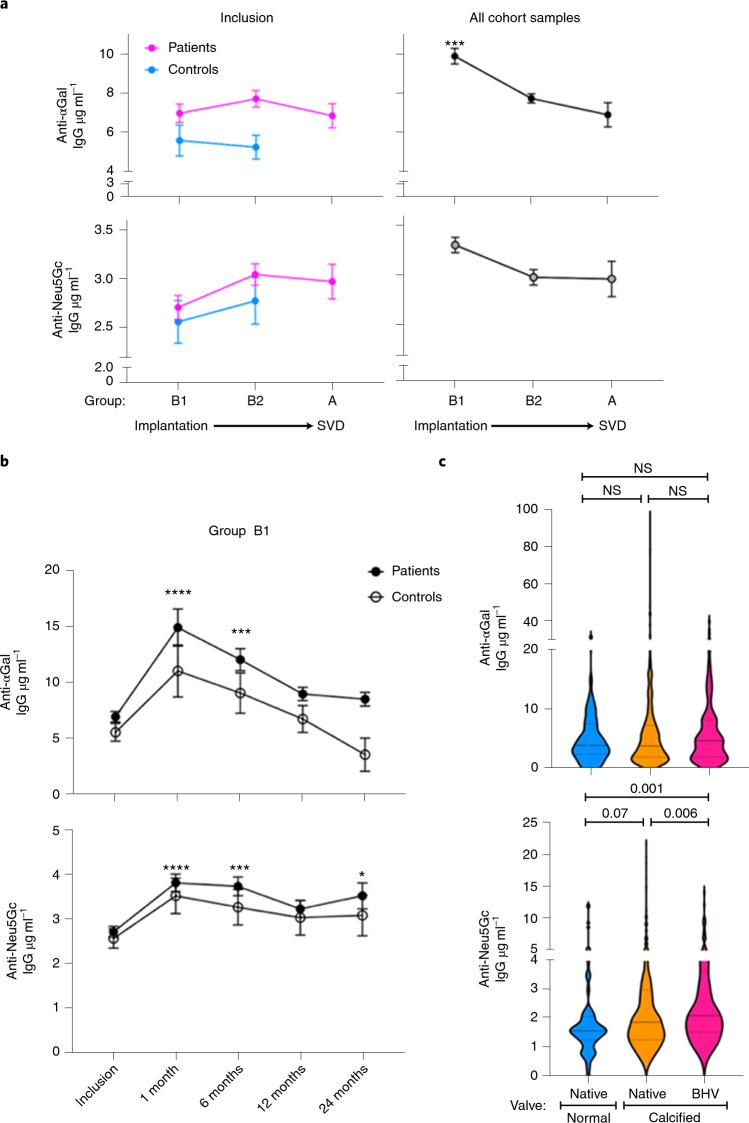
Characteristics and kinetics of anti-αGal and anti-Neu5Gc IgG responses in the Translink cohort. **a**, Anti-αGal and anti-Neu5Gc IgG levels, as measured by ELISA, in the B1, B2 and A groups at inclusion and for the full cohorts (mean ± s.e.m.; anti-αGal/Neu5Gc at inclusion: *n* = 604, 845, 144/605, 874, 170; in full cohort: n = 2,461, 2,344, 144/2,484, 1,860, 170, respectively in groups B1, B2 and A; Kruskal–Wallis test with Dunn’s multiple comparison test; ***P = 0.0004). **b**, Time course of anti-αGal and anti-Neu5Gc IgG in group B1 BHV patients versus controls (mean ± s.e.m.; anti-αGal *n* = 2,007/456 and anti-Neu5Gc *n* = 2,027/459 of BHV/controls, respectively; two-way ANOVA with Dunnett’s multiple comparisons test; anti-αGal, ****P < 0.0001, ***P = 0.0004; anti-Neu5Gc, ****P < 0.0001, ***P = 0.0002, *P = 0.019). **c**, Anti-αGal and anti-Neu5Gc IgG levels in patients with calcified native valves (*n* = 479; group B1 MHV/BHV candidate patients at inclusion) or patients with calcified BHVs (*n* = 170; group A patients at inclusion) compared to patients with normally functioning native valves (*n* = 59; group B1 patients with CABG; Kruskal–Wallis test followed by two-stage linear step-up procedure of Benjamini, Krieger and Yekutieli to correct for multiple comparisons by controlling the FDR < 0.05).

The Translink cohort covers the period from implantation through SVD (Fig. 1a), including patients before implantation (B1) through those with maintained BHV function (B1/B2), and finally those with confirmed SVD (A) and their controls (Fig. 1a, Table 1 and Supplementary Table 1). Anti-αGal IgG was measured by ELISA against αGal (αGal-polyacrylamide (PAA)) and anti-Neu5Gc IgG was measured by enzyme immunoassay (EIA) (against Neu5Gc glycoproteins). Anti-αGal IgG did not correlate with anti-Neu5Gc IgG responses in any of the groups (A, *R*^2^ = 0.111; B1, *R*^2^ = 0.085; B2, *R*^2^ = 0.018). To evaluate the variability in levels of antibodies at inclusion, the baseline measurements of antibodies in BHV/control patients were first compared across the groups. At inclusion, anti-αGal IgG levels were 2.4-fold higher than anti-Neu5Gc IgG (mean at inclusion: 7 ± 0.4 µg ml^−1^ versus 2.9 ± 0.2 µg ml^−1^, respectively; Fig. 2a). For both antibodies there were no significant differences between groups B1, B2 and A, with marginally increased anti-Neu5Gc IgG at B2/A compared to B1 (Fig. 2a).

Before considering disease-related factors, general variability in the levels of antibodies was investigated in the whole cohort across the groups (total patients/controls at inclusion/follow-up). In group B1, anti-αGal IgG was threefold higher than anti-Neu5Gc IgG (B1 mean: 9.8 ± 0.4 µg ml^−1^ versus 3.3 ± 0.1 µg ml^−1^, respectively; Fig. 2a). Furthermore, anti-αGal IgG was significantly higher in group B1 compared to groups B2 and A (Fig. 2a). Anti-Neu5Gc IgG followed a similar but nonsignificant trend (Fig. 2a) showing higher levels in group B1 of de novo BHV recipients within the first two years after implantation and their controls.

Subsequently, we analyzed variability in antibody levels between BHV patients and controls. Monitoring the kinetics of response in group B1 BHV recipients revealed that both anti-αGal and anti-Neu5Gc IgG increased significantly after implantation, peaking after 1 and 6 months (*P* < 0.0001), and remained higher than baseline at 24 months, which is particularly significant for anti-Neu5Gc IgG (Fig. 2b). This was also confirmed by a paired *t*-test analysis comparing levels of anti-Neu5Gc IgG at 24 months versus inclusion, which was significant only in BHV patients (*P* = 0.0085) but not in controls. While a similar increase appeared in the respective controls, it was not statistically significant (smaller sample size in controls compared to BHV patients limited the statistical power; Fig. 2b). Surgery/procedure-related inflammation in both BHV patients and controls may increase antibody responses^25,26^ but BHVs seem to induce further anti-αGal and anti-Neu5Gc IgG.

In the small subgroup of patients receiving BHV through percutaneous implantation without surgery (patients with TAVI, *n* = 71; 14.2% of group B1 patients), there was no significant difference in the kinetics of antibodies compared to surgery-implanted BHV patients with a transient increase in the levels of antibodies after BHV implantation in both groups. Compared to controls (where patients were not exposed to extracorporeal circulation), in univariate analysis, anti-αGal IgG levels were significantly higher before BHV implantation (*P* = 0.008), increased more slowly (*P* = 0.011) then decreased more slowly (*P* < 0.001). Anti-Neu5Gc IgG levels and kinetics were not significantly different between BHV patients without surgery and controls. Thus procedure-related inflammation could possibly be lower in the transcatheter approach than in conventional surgery. However, given the small sample size, direct comparison of the effects of transcatheter versus surgical procedures should be further conducted in a larger cohort.

Antibody responses could not be reliably correlated with BHV deterioration by extended Cox model, although significant correlation was found between severe PPMs (15 out of 500) and early SVD cases (hazard ratio (HR) = 5.5, range 1.1–26.7). However, this association should be interpreted with caution given the low number of patients detected with early signs of calcification (ten BHV patients in group B1) against the large variability of antibody responses. In group B2, there were no significant changes in levels of both anti-Neu5Gc and anti-αGal IgG over the two-year follow-up in long-term BHV recipients versus controls.

Valve construction could potentially influence SVD risk, particularly Mitroflow^5^. Although Mitroflow implantations were limited in the Translink cohort due to removal from the market (4% in group B1), focused analysis on different BHV types (Magna Ease/SAPIEN/Trifecta/Perimount/Mitroflow/SOLO/Perceval, 47, 10, 8, 7, 7, 5 and 4%, respectively) showed that only Mitroflow was significantly associated with SVD in univariate analysis (HR = 2.675, 95% confidence interval (CI) = 1.023–6.995, *P* = 0.045) but not in multivariate analysis. There was no significant effect of BHV biomaterial on antibody responses in BHV recipients (Supplementary Table 4; group B1, *n* = 500 BHV biomaterial bovine/equine/porcine origin, 91.4, 4 and 4.6%, respectively).

To assess the possible contribution of other confounding factors to the kinetics of antibody levels after BHV implantation, group B1 antibody responses were examined through multivariate two-phase linear mixed-effect models (Supplementary Table 5), particularly adjusting for age at inclusion, sex and sample collecting center variables (Table 1). A change in linear slope was seen at 45 d for the anti-αGal IgG model and at 50 d for the anti-Neu5Gc IgG model. At baseline, there was no significant difference between BHV and controls for both anti-αGal and anti-Neu5Gc IgG (*P* = 0.25 and *P* = 0.27, respectively; Supplementary Table 5). The kinetics of anti-αGal IgG showed an initial increase in antibody levels in both controls and BHV patients within 45 d after surgery (phase 1; not significantly different between the groups, *P* = 0.36), then both groups showed a slow decrease, which was significantly faster in controls compared to BHV patients (phase 2; *P* < 0.0001; Supplementary Table 5). Conversely, the kinetics of anti-Neu5Gc IgG were not significantly different between controls and BHV patients (phase 1: *P* = 0.53; phase 2: *P* = 0.17; Supplementary Table 5), which seemed to run in parallel, only with higher levels in BHV patients compared to controls. Altogether, these multivariate analyses show the different kinetics of the levels of anti-αGal and anti-Neu5Gc IgG antibodies after BHV implantation.

**Extended Data Fig. 1:**
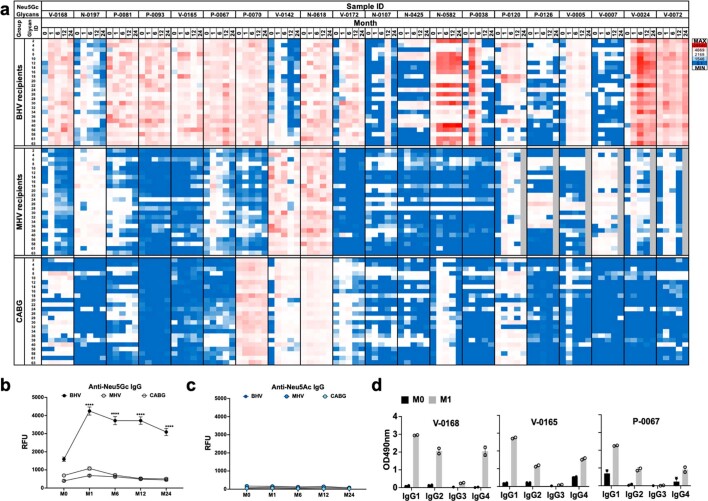
Glycan microarray analysis of representative group B1 patients. **a**, Top 20 responders against Neu5Gc in group B1 (BHV patients and MHV/CABG controls) were examined on glycan microarrays. Top responders were selected based on the highest signal at peak response (one month after surgery), as measured by ELISA (EIA assay). Samples of BHV patients and control patients of mechanical heart valve (MHV) replacement or coronary artery bypass grafting (CABG), were examined on glycan microarrays, then slides were scanned, and relative fluorescence units (RFU) obtained (heatmap of all the examined binding arrays together; red highest, blue lowest and white 50^th^ percentile; supplementary array dataset). **b**, Kinetics of average response by array in BHV patients and control patients (n = 60 patients; 20 per group) against all 24 different Neu5Gc-glycans. Each spot is the average response across all glycans and patients within each group (mean±sem; Mixed-effects analysis, Dunnett’s multiple comparisons test, ****, p < 0.0001). **c**, Kinetics of average response by array in BHV patients and control patients against all 24 different Neu5Ac-glycans (matched to the 24 Neu5Gc-glycans; Supplementary Table 6; P1-P24). **d**, Anti-Neu5Gc antibodies were affinity-purified from three representative sera, at two time points [at inclusion (M0) and at one month after implantation (M1); n = 6]. Total yields were higher in M1 compared to M0 (in M0 vs M1: 750 µl V-0168 serum resulted in 14.9 µg vs 80 µg; 700 µl V-0165 serum resulted in 14 µg vs 25.7 µg; 800 µl P-0067 serum resulted in 1.5 µg vs 33 µg). Levels of human IgG subclasses were evaluated by ELISA showing an increase in IgG in M1 compared with M0, particularly of IgG1 > IgG4 > IgG2 » IgG3 (mean ± SD of duplicates).

Neu5Gc can be conjugated to underlying-glycans by α2–3, α2–6 and α2–8 linkages (Supplementary Table 6), which are recognized by multiple anti-Neu5Gc antibodies. To investigate the antibodies repertoire postimplantation, we used glycan microarrays printed with multiple Neu5Gc glycans and their matched pairs of glycans that contained terminal *N*-acetylneuraminic acid (Neu5Ac) (differs from Neu5Gc only by a single oxygen). The group B1 top 20 responders against Neu5Gc were examined (BHV and controls by EIA at peak response, 1 month after surgery; Extended Data Fig. 1 and supplementary array dataset). Although anti-Neu5Gc IgG detection on the array correlated with EIA^27,28^, the array provided detailed diversity responses against individual Neu5Gc glycans and their matched Neu5Ac glycans. Arrays showed increased anti-Neu5Gc IgG levels starting one month after BHV implantation, even in BHV patients with very low baseline response at inclusion (for example, at inclusion: V-0168, N-0197, V-0142, V-0172; Extended Data Fig. 1a,b), which were significantly higher compared to controls. Increased IgG responses were against multiple Neu5Gc glycans (Extended Data Fig. 1a) and specific since the matching self-Neu5Ac glycans were not recognized (Extended Data Fig. 1c).

Anti-Neu5Gc antibodies were then affinity-purified from the serum samples of three patients at inclusion and one month after BHV implantation. Total yields were higher one month after implantation compared to at inclusion confirming a tenfold increase (9.7 ± 2.5) in the total amount of anti-Neu5Gc antibodies one month after BHV implantation (Extended Data Fig. 1d). Subsequent analysis revealed that all IgG subclasses were increased one month after BHV implantation, in particular IgG1, IgG4 and IgG2, while IgG3 only slightly increased in some patients (one month after BHV implantation compared to at inclusion; Extended Data Fig. 1d). This limited analysis further supported the observed increase in serum anti-Neu5Gc antibodies.

BHV SVD and native aortic valve stenosis are distinct pathological processes, yet both are characterized by valve calcification^6^, the final step of leaflet tissue remodeling and inflammation^29^. We evaluated the immunological responses to leaflet tissue explanted from patients with native aortic valve stenosis before BHV implantation (Fig. 2c). Anti-αGal IgG levels did not significantly vary between patients with calcified native valves and those with normal native valves (Fig. 2c; *P* = NS). However, anti-Neu5Gc IgG levels were somewhat higher in patients with calcified native valves compared to those with normal native valves (Fig. 2c; *P* = 0.07). But in patients with calcified BHVs, anti-Neu5Gc IgG levels were significantly higher compared to both normal and calcified native valves (*P* = 0.001 and *P* = 0.006, respectively; Fig. 2c; average anti-Neu5Gc IgG 2.4 ± 0.3 µg ml^−1^ in control native valves, 2.7 ± 0.1 µg ml^−1^ in calcified native valves and 3 ± 0.2 µg ml^−1^ in calcified BHVs).

### Neu5Gc and anti-Neu5Gc IgG in explanted calcified valves

**Fig. 3:**
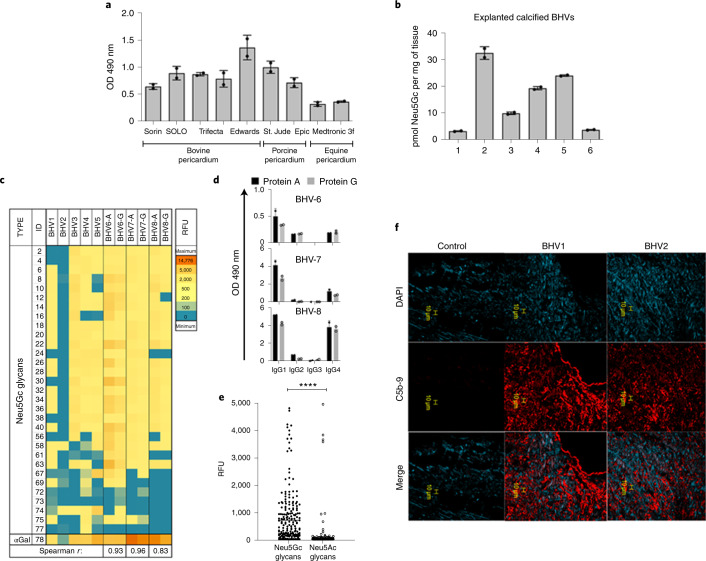
Neu5Gc and anti-Neu5Gc IgG in explanted calcified bioprosthetic heart valves. **a**, Detection of Neu5Gc in homogenates of commercial BHVs by ELISA using chicken anti-Neu5Gc IgY and detection with HRP donkey anti-chicken IgY (mean ± s.e.m.; two different slices from each tissue sample; two independent experiments). **b**, Quantitative analysis of Neu5Gc in calcified BHV explants by DMB–HPLC of BHV homogenates (mean ± s.e.m.; two independent experiments). **c**, Analysis of IgG purified from calcified BHV explants (*n* = 8) by glycan microarrays printed with Neu5Ac/Neu5Gc glycans and αGal. IgG was purified using protein A (BHV-1–BHV-5) or both protein A and protein G (BHV-6–BHV-8). RFU, relative fluorescence unit. **d**, Levels of human IgG subclasses were evaluated by ELISA in IgG purified from the calcified BHV explants by protein A or G (samples BHV-6 to BHV-8 are shown in Fig. 3c; mean ± s.d. of duplicates). **e**, Analysis of IgG binding to Neu5Ac/Neu5Gc glycans by glycan microarrays of IgG purified from calcified BHV explants (*n* = 8; mean ± s.e.m.; each spot represents IgG binding to a specific glycan on the array; two-tailed Mann–Whitney *U*-test, ****P < 0.0001). **f**, IHC of explanted calcified BHVs showing deposition of C5b-9 (representative of two independent experiments; *n* = 2 BHV explants). Scale bar, 10 μm.

**Extended Data Fig. 2:**
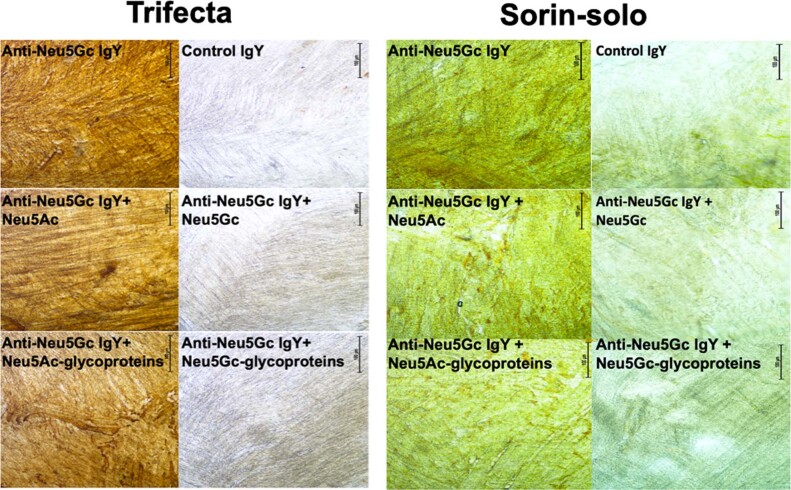
Detection of Neu5Gc on commercial BHVs by immunohistochemistry. Specific detection of Neu5Gc on BHVs by immunohistochemistry of St. Jude Medical Trifecta and Sorin Solo detected with chicken anti-Neu5Gc IgY versus control chicken IgY (n = 2; representative of two independent experiments). Of note, BHV tissue is a dead tissue with extensive pre-treatment such as with glutaraldehyde or other agents (largely undisclosed by each commercial entity). Therefore, the staining seems opaque and different than staining of a living tissue. Neu5Gc staining was inhibited by precomplexing of primary antibody with free Neu5Gc or Neu5Gc-glycoproteins, but not with Neu5Ac or Neu5Ac-glycoproteins, in both stained BHVs.

To expand on limited previous studies^12^, Neu5Gc on BHVs was examined. Neu5Gc was found in homogenates of various commercial BHVs made of porcine, bovine or equine pericardium (Fig. 3a). Of note, tissues from *Cmah*^−/−^ porcine BHVs have no Neu5Gc expression^30^. Immunohistochemistry (IHC) revealed uniform Neu5Gc expression; specific detection was inhibited with free Neu5Gc/Neu5Gc glycoproteins but not with free Neu5Ac/Neu5Ac glycoproteins (Extended Data Fig. 2). These results reinforce previous studies on BVH immunogenicity showing expression of αGal on BHVs^11^. High levels of Neu5Gc were detected in calcified BHVs explanted from human patients (by 1,2-diamino-4, 5-methylenedioxybenzene (DMB)–HPLC, 15.7 ± 4.8 pmol Sia per μg of protein; *n* = 6, mean ± s.e.m.; Fig. 3b), further demonstrating its expression on BHVs even years after implantation. Given that Neu5Gc levels in unused commercial BHVs have been recorded at 193.8 ± 167.8 pmol Neu5Gc per μg of protein^12^, it seems that explanted BHVs retain 19 ± 12% of the initial Neu5Gc content. Thus, Neu5Gc is an integral component of BHVs, both preimplantation and postimplantation.

Highly prevalent αGal and Neu5Gc glycans on BHVs are likely to be recognized by circulating human antibodies^12,17^. To explore this in human BHV recipients, eight explanted calcified BHVs were homogenized, then IgG antibodies were purified and examined on glycan microarrays. To confirm coverage of all IgG subclasses, antibodies were purified by both proteins A and G. There was detectable anti-αGal and weaker anti-Neu5Gc IgG response in all analyzed samples, with variable recognition of Neu5Gc glycans (Fig. 3c). In addition, there was no significant difference in reactivity (Fig. 3c; BHV-6 to BHV-8; nonparametric Spearman correlation) or subclasses between IgG purified by protein A or protein G (BHV-6 to BHV-8), showing mostly IgG1, IgG4 and IgG2 but not IgG3 (Fig. 3d). Of note, the affinity-purified anti-Neu5Gc antibodies from the patient serum samples showed similar distribution of IgG subclasses (Extended Data Fig. 1d). These antibodies recognized multiple Neu5Gc glycans with minimal recognition of Neu5Ac glycans (Fig. 3e and Supplementary Table 6), similar to the serum microarray analysis of group B1 BHV patients (Extended Data Fig. 1). Complement activation is key to antibody‐triggered damage in allo/xenotransplantation^31^, which is characterized by formation C5b-9-containing membrane attack complexes^32^. IHC of explanted calcified BHVs revealed C5b-9 staining (Fig. 3f), suggesting local complement activation that could support a pro-inflammatory microenvironment.

**Extended Data Fig. 3:**
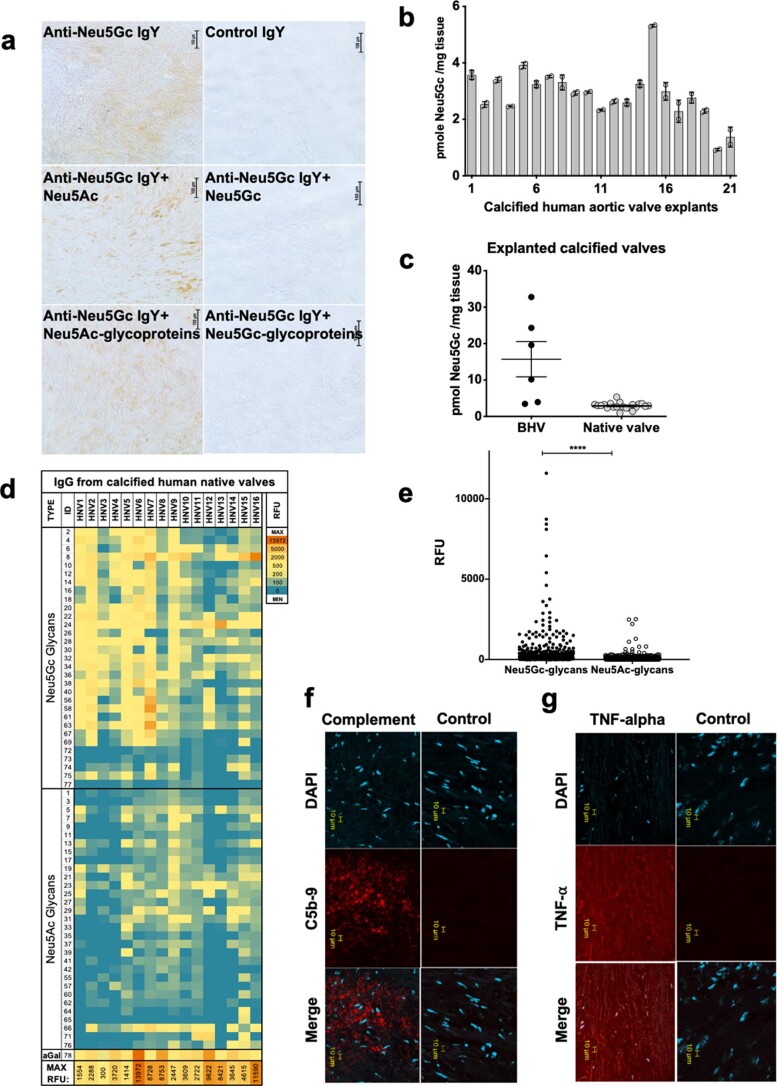
Neu5Gc and anti-Neu5Gc IgG in explanted calcified native aortic human heart valves. **a**, Immunohistochemistry of explanted calcified native human aortic heart valve tissues revealed specific detection of Neu5Gc, by staining with chicken anti-Neu5Gc IgY versus control chicken IgY. Neu5Gc staining was inhibited by precomplexing of primary antibody with free Neu5Gc or Neu5Gc-glycoproetins, but not with Neu5Ac or Neu5Ac-glycoproetins (representative of at least two independent experiments). **b**, Quantitative analysis of Neu5Gc in explanted calcified native aortic valves by DMB-HPLC (n = 21 explants; mean±sem; two independent experiments). **c**, Neu5Gc DMB-HPLC quantification shows much higher Neu5Gc levels in explanted calcified BHVs compared to explanted calcified native valves (mean±sem; n = 6, 21 BHV and native valve explants, respectively). **d**, IgG antibodies were purified from homogenized calcified native valve explants (n = 16) by protein A, then analyzed by glycan microarray printed with αGal and Neu5Gc/Neu5Ac-glycans and relative fluorescence units (RFU) determined after scanning, showing anti-αGal IgG and anti-Neu5Gc IgG response in all analyzed samples. **e**, Glycan microarrays analysis of IgG purified from explants (n = 16) revealed strong recognition of Neu5Gc-glycans while minimal recognition of Neu5Ac-glycans (mean±sem; each spot is IgG binding to a specific glycan on the array of all BHV samples analyzed; Two-tailed Mann-Whitney test, ****, p < 0.0001). **f**, Immunohistochemistry of explanted calcified native valves with mouse-anti-human-C5b-9 IgG shows staining of membrane attack complex (representative of three independent experiments). g, Immunohistochemistry of explanted calcified native valves with biotinylated-rabbit-anti-human TNF-α, detected with Cy3-streptavidin, shows deposition of TNF-α (n = 1).

Dietary Neu5Gc can incorporate onto human cell surfaces^33,34^; thus, we analyzed Neu5Gc and antibodies in explanted calcified native aortic valves. Neu5Gc was found in every calcified native human aortic valve explanted during valve replacement surgery by IHC (Extended Data Fig. 3a) and DMB–HPLC (*n* = 21; Extended Data Fig. 3b). However, Neu5Gc in explanted calcified BHVs was much higher than that found in explanted calcified native valves (15.7 ± 4.8 versus 2.9 ± 0.2 pmol sialic acid per mg of tissue, respectively; Extended Data Fig. 3c). Subsequently, IgG was purified by protein A from Neu5Gc-expressing calcified native valves (*n* = 16), then analyzed by glycan microarrays, revealing some recognition of Neu5Gc glycans in all explants (Extended Data Fig. 3d), with minimal recognition of the paired Neu5Ac glycans (Extended Data Fig. 3e). In addition, IgG recognition of αGal that was printed on the same glycan microarrays was also noted. IHC revealed membrane attack complexes (Extended Data Fig. 3f) and tumor necrosis factor-α (TNF-α) in native calcified aortic valves (Extended Data Fig. 3g). Thus, Neu5Gc/anti-Neu5Gc IgGs were associated with calcification possibly contributing to disease process in native valves.

### Anti-Neu5Gc IgG-mediated calcification in *Cmah*^−/−^ mice

**Fig. 4:**
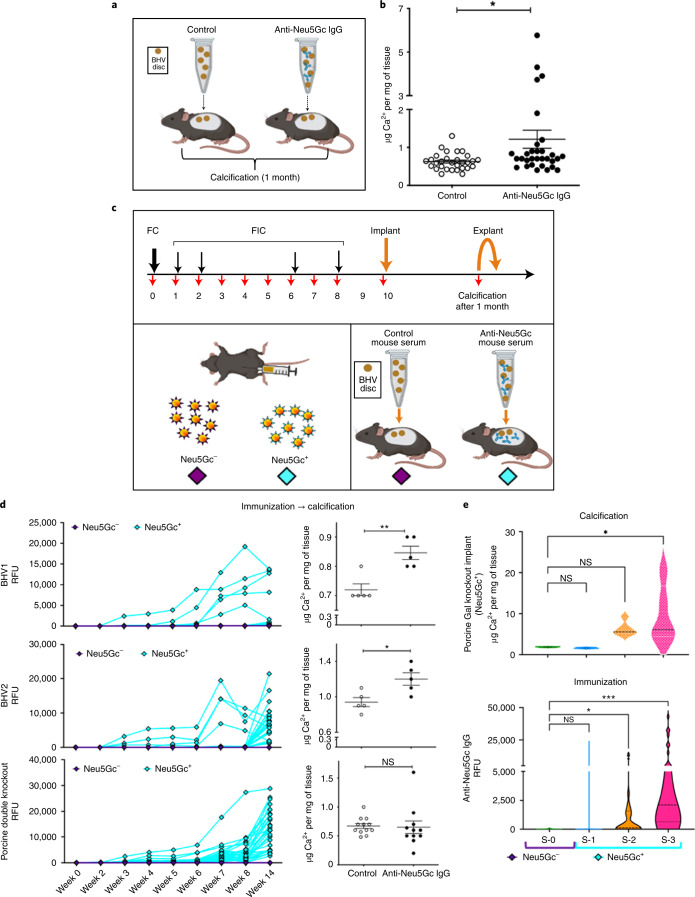
BHV calcification mediated by anti-Neu5Gc IgG in Neu5Gc-deficient *Cmah*^−/−^ mice. **a**, Diagram of the experiment carried out to examine antibody-mediated calcification of BHV in naïve mice. BHV tissue discs (6 mm) were preincubated with affinity-purified human anti-Neu5Gc IgG or PBS carrier control, then implanted into Neu5Gc-deficient mice (*Cmah*^−/−^); the calcium in discs was measured after one month by atomic absorption spectrometry. **b**, Calcium deposition quantified in explanted discs (mean ± s.e.m.; two-tailed nonparametric Mann–Whitney *U*-test; *P* = 0.0134; *n* = 5 BHVs; 2 groups (control/treatment), *n* = 8 mice per group; implanted 4 BHV discs per mouse) **c**, Diagram of the experiment carried out to examine antibody-mediated calcification of BHVs in immunized *Cmah*^−/−^ mice. Mice were immunized with Neu5Gc^−^/Neu5Gc^+^ glyconanoparticles (purple/light blue); then, the immunized-serum pretreated BHV discs were implanted and calcium was quantified after one month. **d**, Anti-Neu5Gc IgG (light blue) or control IgG (purple) responses in immunized mice were confirmed by glycan microarray analysis at the indicated time points (immunization; left panel; two-way ANOVA with Bonferroni multiple comparisons test, *P* < 0.0001); then, tissues discs were implanted in preimmunized mice and calcium levels were measured in explanted discs (calcification; right panel; *n* = 3 tissues of BHVs or porcine αGal/Neu5Gc-deficient (*Ggta1*^−/−^/*Cmah*^−/−^; porcine double knockout); *n* = 8 mice; 5 discs per mouse; mean ± s.e.m.; unpaired two-tailed *t*-test; BHV-1, St Jude Medical Epic porcine aortic valve, *P = 0.0175; BHV-2, Carpentier–Edwards bovine pericardium, **P = 0.0032). Glutaraldehyde-treated porcine double knockout pericardium was similarly implanted in preimmunized mice and calcification measured (mean ± s.e.m.; immunization: two-way ANOVA with Bonferroni multiple comparisons test, *P* < 0.0001; calcification: unpaired two-tailed *t*-test, NS, *P* = 0.0998). **e**, Neu5Gc-containing porcine pericardium (*Ggta1*^−/−^; Gal knockout; glutaraldehyde-treated) was implanted into control Neu5Gc^−^-immunized mice (*n* = 2; S-0) or into Neu5Gc^+^-immunized mice that had low or high anti-Neu5Gc IgG response according to glycan microarray analysis (*n* = 3; low: S-1, S-2; high: S-3); then, calcification was measured after 1 month (4 discs per mouse; thick broken line, median; mixed-effects analysis with Dunnett multiple comparisons test; immunization: *P = 0.0107, ***P = 0.0008; calcification: *P = 0.0183).

**Extended Data Fig. 4:**
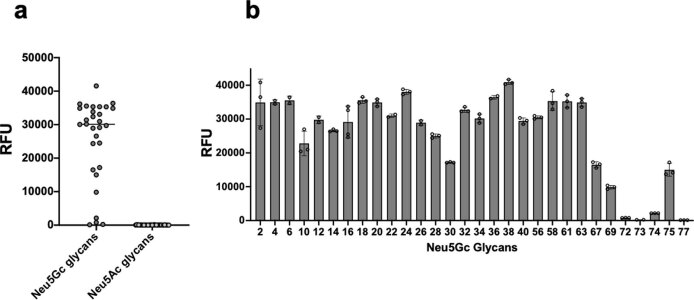
Affinity-purified human anti-Neu5Gc IgG are highly specific. **a-b**, Anti-Neu5Gc antibodies were affinity-purified from pooled human IgG (IVIG, Privigen) over columns containing Neu5Ac/Neu5Gc-glycoproteins (representative of two independent experiments). Binding of purified anti-Neu5Gc IgG was analyzed by sialoglycan microarray and relative fluorescence units (RFU) determined after scanning showing bindings to Neu5Gc-glycans but not Neu5Ac-glycans (a) at a diverse pattern of recognition (b; mean ± SD).

Subcutaneous implantation models have been widely used to study calcification of bioprosthetic tissues^35,36^, particularly to investigate the role of αGal/anti-αGal, concluding that anti-αGal antibodies accelerate calcification of wild-type (WT) but not αGal-deficient glutaraldehyde-fixed porcine pericardium^8,9^. To similarly assess the role of Neu5Gc/anti-Neu5Gc antibodies in the calcification process, a wide collection of commercial BVHs of porcine, bovine and equine pericardial tissues were implanted subcutaneously in Neu5Gc-deficient mice (*Cmah*^−/−^; mimicking the dysfunctional *CMAH*/*CMAHP* gene in humans), in the presence or absence of human anti-Neu5Gc IgG (Fig. 4a). Human anti-Neu5Gc IgG was affinity-purified from pooled human IgG and its specificity confirmed by glycan microarray (Extended Data Fig. 4). Tissue discs of commercial BHVs were incubated with these human anti-Neu5Gc IgG or carrier control, followed by their subcutaneous implantation in *Cmah*^−/−^ mice; after one month, the calcification of explanted BHV discs was measured by atomic absorption spectrometry (Fig. 4a). Higher calcium levels were found in discs treated with anti-Neu5Gc IgG compared to control-treated discs (Fig. 4b), supporting induced BHV calcification by anti-Neu5Gc IgG.

Humans already have circulating anti-αGal and anti-Neu5Gc IgG^17,37,38^, including before BHV implantation (Fig. 2a). Therefore, in a second experimental setup, we studied BHV calcification in the presence of circulating mouse anti-Neu5Gc antibodies by subcutaneous implantation in preimmunized mice recipients (Fig. 4c). *Cmah*^−/−^ mice were first immunized with Neu5Gc-containing nano-ghost glyconanoparticles (Neu5Gc^+^) or controls immunized with Neu5Ac-containing glyconanoparticles (Neu5Gc^−^)^39^ (Fig. 4c). A specific elicited anti-Neu5Gc IgG response was observed by glycan microarrays only in the Neu5Gc^+^-immunized mice (Fig. 4d, immunization). Sera collected from immunized mice were then cleared from antibodies against the carrier for subsequent studies.

Tissue discs were preincubated with mouse anti-Neu5Gc sera or control-immunized sera, then these discs were implanted into mice that either had or lacked serum anti-Neu5Gc antibodies (Neu5Gc^+^-immunized mice or control Neu5Gc^−^-immunized mice, respectively; Fig. 4c,d). Implants were collected after one month and calcification was measured. Both BHV samples (BHV-1: bovine pericardium; BHV-2: porcine aortic valve) showed higher calcification in the presence of serum anti-Neu5Gc IgG (Fig. 4d). In contrast, there was no increase in calcification in implants of porcine pericardial tissues lacking both αGal and Neu5Gc (porcine double knockout from genetically engineered *Ggta1*^−/−^/*Cmah*^−/−^ porcine^40^), despite the presence of high levels of serum anti-Neu5Gc IgG (Fig. 4d). In Neu5Gc-expressing porcine pericardial implants (porcine Gal knockout; *Ggta1*^−/−^ porcine^41^), increased calcification was mediated by higher anti-Neu5Gc IgG levels (Fig. 4e; S-3), while no antibodies or low levels did not support calcification (Fig. 4e; S-0, S-1 and S-2). Altogether, coexisting Neu5Gc/anti-Neu5Gc IgG contributed to BHV calcification and suggested that removal of both αGal and Neu5Gc by genetic engineering is promising for decreasing or preventing calcification and eventual SVD.

## Discussion

The role αGal/anti-αGal IgG in BHV calcification has been studied in mice^7–9^ and humans^7,10^, yet Neu5Gc/anti-Neu5Gc IgG has not been fully investigated. In mice, elicited anti-Neu5Gc antibodies exacerbate chronic inflammation-mediated diseases^38,42–44^ by xenosialitis^38,43,45^. While xenosialitis in human patients is controversial^34,46^, there is compelling evidence that an immune response against αGal or Neu5Gc antigens is detrimental in a xenogeneic context. Translink investigated approximately 5,000 serum samples for humoral responses against implanted aortic animal-derived BHVs, particularly anti-αGal and anti-Neu5Gc IgGs, up to approximately 15 years postimplantation.

Our data show that BHVs are immunogenic and elicit a concomitant increase of anti-αGal and anti-Neu5Gc IgGs in the first six months after BHV implantation. We also noted nonsignificant increased antibody levels in controls (fewer than BHV patients). This suggests that surgical or extracorporeal circulation procedures (for example, cardiopulmonary bypass (CPB)) contribute to antibody increase in both BHV-implanted patients and controls. There is compelling evidence from clinical/experimental studies that surgical procedures can trigger inflammation. Cardiac surgery or extracorporeal circulation results in increased levels of cytokines in the blood, particularly TNF-α and interleukin-6 (ref. ^26^), that could contribute to the nonspecific effect of increased serum antibodies. Three months after CPB, the serum levels of anti-cytomegalovirus IgG antibody titers were elevated in patients and these were associated with the macrophage-like characteristics of circulating monocytes^47^. Thus, reducing inflammatory effects, particularly immediately after surgery, could potentially lower antibody levels and perhaps reduce their possible deleterious effects on implanted BHVs.

Generally, anti-αGal IgG levels were 2.5 to threefold higher than those of anti-Neu5Gc IgG; each antibody had different kinetics postimplantation (even after adjustment for various confounding factors such as age, sex and hospital) and their levels did not correlate, as reported in other clinical contexts^48,49^. While both types of antibodies increased after BHV implantation, anti-αGal IgG levels dropped more rapidly during follow-up, whereas anti-Neu5Gc IgG levels remained high longer after the initial increase, indicating longer exposure of implanted valves to anti-Neu5Gc IgG. Thus, anti-αGal IgG may play a more crucial role early after implantation, whereas anti-Neu5Gc IgG could have an additional long-term impact.

Long-lasting anti-Neu5Gc IgG responses have been shown in patients treated with Neu5Gc-glycosylated biotherapeutics, even after only a short exposure^48,50,51^. All humans are immunologically primed for Neu5Gc from the diet^28^ and for αGal through continuous stimulation by αGal-expressing gut microbiota^52,53^. Due to this continuous exposure to antigens, BHV implantation likely elicits an additional secondary immune response that further supports high anti-αGal IgG and sustains anti-Neu5Gc IgG, with variability in IgG repertoire over time and between patients.

Higher Neu5Gc levels were found on explanted BHV compared to native valves (approximately fivefold). Neu5Gc found in calcified native aortic valves explanted from human patients represented only trace deposition of dietary Neu5Gc on these tissues compared to the levels detected on commercial BHVs. This diet-derived Neu5Gc expression could be mediated by the pro-angiogenic properties of human aortic valve interstitial cells during native calcific aortic valve disease^54^.

In agreement with the presence of Neu5Gc and αGal antigens on BHVs, anti-αGal and anti-Neu5Gc IgGs could also be detected and eluted from calcified BHVs. Given the high specificity of the detection assay, our study provides direct evidence for tissue distribution of Neu5Gc on BHV, despite devitalization procedures during production (for example, glutaraldehyde fixation/formaldehyde/phospholipid reduction)^24^. Years after implantation, approximately 20% of Neu5Gc was still found in explanted calcified BHVs together with localized anti-Neu5Gc and anti-αGal IgGs. Limited analyses revealed that anti-Neu5Gc IgG were IgG1, IgG4, IgG2 but not IgG3. Complement deposition was found in both calcified valve tissues and TNF-α was detected in calcified native valves. Of note, complement can be activated in stenotic native aortic valves^55^, suggested to be involved in structural deterioration of bovine pericardial BHV^56^, and native aortic valve calcification can be influenced by inflammation involving TNF-α^57^.

Unlike native valves, BHVs lack valve interstitial cells that produce and remodel the extracellular matrix^58^. However, it was recently suggested that the pathogenesis of BHV SVD could also be mediated by host immune responses, through processes that resemble long‐term transplant rejection, atherosclerosis and calcification of native aortic valves^59^. While native aortic valve stenosis and BHV SVD demonstrate considerable differences^59^, they both share leaflet tissue remodeling and inflammation^29^ that finally result in leaflet calcification^6^, presumed to be associated with local immunological responses^59^. Some of our data suggest common mechanisms for SVD and native valve disease that warrants further dedicated studies. However, this concept faces major differences in levels of antigens in native valves compared to fresh xeno-derived BHVs (approximately 12–18-fold higher in BHV; 15.7 ± 4.8 versus 193.8 ± 167.8 pmol Neu5Gc per mg of tissue, respectively). In addition, other unexplored xenogeneic antigens on BHV tissue could contribute to immunogenicity of animal-derived implants and possibly elicit antibodies, which could also support valve calcification^60^. For example, the Sd(a) blood group glycan is another carbohydrate xenoantigen of potential reactivity on BHVs and prompted the generation of triple knockout donor animals that lack expression of αGal, Neu5Gc and Sd(a) (knockout glycosyltransferases encoding the genes *GGTA1*, *CMAH* and B4GALNT2, respectively)^60^.

Finally, our studies in Neu5Gc-deficient mice demonstrated the role of Neu5Gc/anti-Neu5Gc IgG in mediating increased calcification that could possibly depend on the actual levels of circulating antibodies. Importantly, tissues that lack αGal and Neu5Gc carbohydrate xenoantigens did not trigger antibody-mediated calcification, providing hope for safer next-generation bioprosthetic heart valves. Studies in mice have some limitations, particularly since in humans anti-αGal IgG or anti-Neu5Gc IgG responses could be affected by factors other than the glycans on the BHV device itself (that is, microbiota or diet, respectively), which likely also contribute to these humoral response kinetics.

Our study has several limitations. The heterogeneity of the Translink cohort limits interpretability of certain aspects due to low occurrence of events. Based on 4,998 blood samples from 1,668 patients and a survey of 34 ± 43 months, we found that early signs of calcification or SVD can be detected in some patients already within the first 2 years after implantation by CT scans or echocardiography; however, reliable direct correlation analysis with immune responses or prosthesis size could not be achieved given their low incidence. Of note, our data provide only a limited picture of the humoral immune response against BHV (mainly bovine pericardium) and could not assess the local cellular response. In addition, this study did not account for anticoagulation/antiplatelet treatment in individual patients, which could affect SVD.

Altogether, these preclinical and clinical studies support the hypothesis that αGal/anti-αGal IgG and Neu5Gc/anti-Neu5Gc IgG mediate events that lead to eventual BHV calcification and deterioration. These studies justify new extensive clinical studies into engineered BHVs lacking major glycan xenoantigens, aiming at decreasing SVD incidence, to allow widening use of such safer nonimmunogenic BHVs to younger patients. Other Translink studies focus on the development of preventive and therapeutic strategies to combat BHV-related adverse immune reactions, including production of next-generation immune-resistant BHV that lack expression of putative immunodominant epitopes, which are potentially responsible for premature graft failure. In addition, Translink is engaged in the generation of specifically designed molecules to protect implants, and possibly BHV recipients, from immune-mediated damage. Finally, this ‘real-life’ very unique large cohort, despite its complexity, can be used as an important resource and/or a starting point for further studies.

## Methods

### Materials

The following antibodies were obtained from BioLegend: purified polyclonal chicken anti-Neu5Gc IgY (catalog no. 146903, 1 μg ml^−1^ or 10 μg ml^−1^); control IgY (catalog no. 402101, 10 μg ml−1). The following antibodies were obtained from Jackson ImmunoResearch: AffiniPure donkey anti-chicken IgY (IgG) (H+L) (1:3,000 or 1:500 dilution; catalog no. 703-005-155); Cy3-AffiniPure goat anti-human IgG (H+L) (catalog no. 109-165-003, 1.5 μg ml^−1^); AffiniPure goat anti-mouse IgG, Fcγ fragment specific (catalog no. 115-005-008, 7.5 μg ml^−1^); peroxidase AffiniPure goat anti-mouse IgG, Fcγ fragment specific (1:5000 dilution; catalog no. 115-035-071); peroxidase AffiniPure F(ab′)_2_ fragment goat anti-human IgG, Fcγ fragment specific (1:1,000 dilution; catalog no. AB_2337596); ChromPure human IgG, whole molecule (1:1,000 dilution; catalog no. 009-000-003); Cy3 streptavidin (catalog no. 016-160-084, 5 μg ml^−1^). The following antibodies were obtained from Invitrogen: mouse anti-human IgG1 Fc (clone HP6069, 1:300 dilution; catalog no. A-10648); mouse anti-human IgG2 horseradish peroxidase (HRP) (clone HP6014, 1:300 dilution; catalog no. 05-0520); mouse anti-human IgG3 (Hinge) (clone HP6047, 1:300 dilution; catalog no. 05-3620). The following antibodies were obtained from Thermo Fisher Scientific: mouse anti-human IgG4 HRP (clone HP6025, 1:300 dilution; catalog no. MA1-34437). Biotinylated rabbit polyclonal anti-human TNF-α antibody was obtained from PeproTech (catalog no. 500-P31A, 0.1 μg ml^−1^) and mouse monoclonal antibody against human C5b-9 was obtained from Abcam (clone aE11, 10 μg ml^−1^; catalog no. AB66768). All antibodies were used at optimized saturating conditions. Avidin/biotin blocking kit was obtained from Vector Laboratories). DAPI, Fluoromount Aqueous Mounting Medium and collagenase type 2 were obtained from Sigma-Aldrich. Protein A Sepharose 4 Fast Flow beads were obtained from GE Healthcare (Sigma, GE17-5280-01). The Neu5Gc carbohydrate was obtained from Inalco and the Neu5Ac carbohydrate from Nacalai Tesque. The αGal antigen was obtained from Lectinity (Galα1-3Galβ-OCH_2_CH_2_CH_2_NH_2_ PAA conjugate of approximately 20 kDa; B_di_-C3-PAA; 5 μg ml^−1^; catalog no. 0088).

### Ethics for clinical studies

The study protocol was reviewed and approved by the European Commission Seventh Framework Programme (FP7) Ethics Committee and the ethics committees of the five hospitals participating in sample collection: the Nantes University Hospital in France; the Bellvitge University Hospital (HUB-ICS) of the Catalan Health Institute in Barcelona, Spain; the University Hospital Vall D’Hebron (HUVH-ICS) of the Catalan Health Institute in Barcelona, Spain; the Azienda Ospedaliera di Padova in Padova, Italy; and St Boniface Hospital in Winnipeg, Canada. All clinical investigations were conducted according to the principles expressed in the Declaration of Helsinki (2013). All study participants provided written informed consent to participate in the trial. Patients’ samples were shipped to investigational laboratories for immunological studies and used in accordance with the Declaration of Helsinki and the Institutional Review Boards of Tel Aviv University and the University of Padova Hospital.

### Translink study design

Translink is a European (and Canadian), prospective, multicenter, open study (clinical trial number NCT02023970), designed to evaluate the kinetics of the immune response before and after implantation of aortic BHVs up to approximately 15 years posttreatment. Patients were enrolled into four groups: (1) group A: BHV recipients with confirmed SVD; (2) group B1: de novo BHV recipients enrolled before AVR with follow-up after 1, 6, 12 and 24 months; (3) group B2: patients with implanted BHVs performed at least 4 years before inclusion, with follow-up after 12 and 24 months; and (4) control B1/B2 groups of non-BHV-implanted patients who received MHVs or patients who required a CABG (Fig.1a and Table 1; Supplementary Table 1 describes the inclusion and exclusion criteria). Enrolled patients provided blood samples and were monitored by echocardiography (Fig. 1c).

### Study participants

Supplementary Table 1 describes the inclusion and exclusion criteria for patients enrolled in groups A, B1 and B2.

#### Group A

A total of 174 patients with an SVD diagnosis were prospectively enrolled between September 2013 and December 2017. Patients were considered eligible for the study if they presented SVD defined by echocardiography with a mean transvalvular gradient ≥30 mmHg and effective orifice area (EOA) ≤ 1 cm^2^ worsening over time or intra-prosthetic insufficiency greater than grade 2/4 and leaflet tissue alteration. Patients were enrolled into the trial by cardiologists at each investigational site. Group A patients were diagnosed with SVD and enrolled in the study 8.4 ± 3.4 years after surgery (minimum-maximum: 2.3–21.6), at the age of 77.0 ± 10.5 years.

#### Group B1

Five hundred patients planned for BHV replacement in 4 European centers were prospectively enrolled between January 2014 and June 2016. Patients were considered eligible for the study if they received only one surgical or percutaneous aortic BHV, either isolated or associated with other procedures such as CABG (*n* = 95), mitral or tricuspid valve repair (*n* = 19), radiofrequency (*n* = 13) and Bentall (*n* = 11). Excluded patients were those who received an immunosuppressive regimen before and/or after BHV implantation, who underwent previous cardiac surgery or previous percutaneous BHV implantation or who needed more than one BHV implantation. The B1 control group (non-BHV) consisted of 118 patients planned for MHV replacement or CABG. Group B1 BHV and control non-BHV patients were enrolled into the trial by cardiac surgeons and/or cardiologists at each investigational site.

#### Group B2

Group B2 patients were enrolled during the same period between January 2014 and June 2016 and included 752 patients who underwent BHV replacement at least 4 years before enrollment at each center. Patients were considered eligible for the study if they received BHV replacement alone or associated with other procedures such as CABG (*n* = 193), mitral or tricuspid valve repair (*n* = 31), radiofrequency (*n* = 27) and Bentall procedure (*n* = 19). The B2 control group included 124 patients who underwent MHV implantation or CABG at least 4 years before enrollment. Preoperative and early postoperative measures were collected retrospectively based on surgical reports and preoperative/postoperative echocardiographic examinations. No blood sample was available for the preoperative immunological analyses. Group B2 BHV patients and controls were contacted by phone by a clinical research associate, then enrolled into the trial by cardiologists at each investigational site.

Of note, groups A and B2 were determined according to baseline echocardiography. Patients were either in group A or B2 but not in both groups. At baseline, a patient with confirmed SVD was enrolled in group A. Group B2 patients who developed SVD during follow-up remained in group B2.

Some patients missed the follow-up visits (Supplementary Table 3) due to either patient/medical resource not being available, economic or logistical problems. Missing visits were especially high in group B2, which was constituted slowly so the follow-up time was too short to schedule a second visit after one or two years for some of them. Group B2 patients were also older (78 ± 8) compared with group B1 patients (74 ± 8); therefore, many of these patients did not want or were not able to come back for a second visit. Regarding group B1, missing visits at 24 months were also related to a short follow-up period after inclusion. In both groups B1 and B2, BHV patients were 8–10 years older and with a larger proportion of women than in controls, although in both groups most patients were men. There were more individuals with a history of smoking in controls compared to patients in group B1 but not in group B2. Most patients received BHVs via surgery (B1: 85.8%; B2: 99.2%) rather than TAVI; in the control groups, most patients underwent MHV surgery (B1: 51.7%; B2: 65.3%) rather than CABG (Table 1). Of note, a higher number of deaths were recorded in group B2 (*n* = 34, 4.5%) compared to group B1 (*n* = 4, 0.8%), which is partly explained by an older age (78 ± 8 versus 74 ± 8 years) at inclusion.

### Description of implanted BHVs in the Translink cohort

In the Translink study, the choice of BHV type was left to the decision of the cardio-surgical team of each center. Patients were implanted with the most frequently implanted BHVs worldwide: two types of surgical porcine valves: Mosaic or Hancock II (Medtronic); 6 different surgical bovine pericardium valves: Perimount Carpentier–Edwards (Edwards Lifesciences), Magna Ease (Edwards Lifesciences), Trifecta (St Jude Medical), Perceval or SOLO bioprostheses (Sorin Biomedica Cardio) and the Mitroflow PRT valve (Sorin Biomedica Cardio); one surgical equine valve: 3F Valve Enable (Medtronic) and two percutaneous pericardium valves (TAVI): CoreValve (porcine pericardium; Medtronic) and SAPIEN valve (bovine pericardium; Edwards Lifesciences). The operating techniques and surgical valve model selection were left to the operating surgeon’s discretion. The distribution of types of implanted biological prostheses in groups B1/B2 is detailed in Fig. 1b.

### Clinical assessment of the Translink cohort

#### Group A

A total of 174 patients with SVD were assessed once at the time of SVD diagnosis. The baseline assessments included clinical evaluation and blood collection for immunological analyses (Fig. 1a).

#### Group B1

BHV patients/controls were assessed preoperatively (baseline), then at 1, 6, 12 and 24 months of follow-up (Fig. 1a and Fig. 1c). The preoperative and follow-up assessments included clinical evaluation and blood collection for immunological analyses. The group B1 patient follow-up was undertaken during on-site clinical visits at each participating center. The mean follow-up of B1 patients was 21 ± 5 months after BHV implantation.

#### Group B2

BHV patients/controls were assessed at inclusion (baseline) and at 12 and 24 months of follow-up (Fig. 1a and Fig. 1c). The baseline and follow-up assessments included clinical evaluation and blood collection for immunological analyses. The mean delay from implantation of B2 patients was 104 ± 22 months. Patient follow-up was undertaken during clinical visits at each participating center.

Of note, the policy regarding anticoagulation/antiplatelet treatment after valve implantation was the same in all Translink centers (vitamin K antagonist in patients with mechanical valves; vitamin K antagonist or other oral anticoagulant in patients with atrial fibrillation; antiplatelet treatment by aspirin alone after surgical bioprosthesis implantation; antiplatelet treatment by aspirin and clopidogrel after surgical TAVI; some patients with anticoagulation were also under aspirin treatment for atherosclerosis such as coronary artery disease).

### BHV assessment of Translink cohort by echocardiography and CT scan

Echocardiography examinations were carried out and analyzed by experienced investigators (C.C., N.P., T.L.T., A.R., A.E. and L.B.) on commercial ultrasound systems (GE Vivid Series) and stored (ImageVault 5.0 and Echopac software 12.1–13.1) in a centralized CoreLab (Nantes, France). In group B1 patients, echocardiography was carried out for all patients at baseline and in all available patients at 6 months and between 12 and 24 months after BHV replacement. At the 21 ± 5 months follow-up, echocardiographic evaluation was available in 371 patients (92.8% out of 400 alive) (Supplementary Table 2). Briefly, the diameter of the left ventricular outflow tract was carefully measured in the parasternal long axis view, the velocity time integral of the left ventricular outflow tract and the aortic velocity time integral were measured in the apical three- or five-chamber view with pulsed and continuous wave Doppler, respectively. The BHV dimensionless index and EOA were calculated. In addition, PPM was defined postoperatively using the reference values of the indexed EOA as published previously^61^: the PPM was considered moderate if the indexed EOA was >0.65 cm^−^^2^ m^−^^2^ but ≤0.85 cm^−^^2^/m^−^^2^ and severe if it was ≤0.65 cm^−^^2^/m^−^^2^. The left ventricular ejection fraction was measured using the biplane Simpson method. During follow-up, early SVD was defined according to current recommendations^62,63^ as an increase in mean gradient >10 mmHg or the worsening of at least 1 grade of intra-prosthetic aortic regurgitation and abnormal leaflet aspect. In a subgroup of B1 patients (Nantes, France), a CT evaluation of BHV was carried out at the time of the last echocardiography between 12 and 24 months of follow-up to assess the calcium scoring of leaflets. A calcium score >0 was considered abnormal.

### Measurement of anti-αGal IgG by ELISA

Human serum samples were obtained from the Translink clinical centers and used in accordance with the Declaration of Helsinki and University of Padova Hospital Institutional Review Board. Specific overall anti-αGal reactivity in human sera was evaluated while blinded to patient/control status. Anti-αGal IgG ELISA was adapted from Scobie et al.^50^. Briefly, polystyrene microtiter plates (NUNC Medisorp; Thermo Fisher Scientific) were coated with Galα1-3Galβ-PAA conjugate (5 µg ml^−1^, Bdi-C3 PAA) in 0.1 M carbonate buffer, pH 9.6, overnight at 4 °C. The plates were then washed with 0.1 M PBS, pH 7.4, containing 0.1% Tween 20 and blocked with PBS/0.5% fish gelatin (Sigma-Aldrich) for 4 h at 4 °C. Human sera (50 µl, tested in triplicate wells at a dilution of 1:320 and 1:640 for each anti-αGal IgG determination in 0.1 M PBS, pH 7.4, containing 0.5% Tween 20) were incubated for 1 h at 4 °C. Subsequently, 50 µl of peroxidase AffiniPure F(ab′)_2_ goat anti-human IgG diluted 1:1,000 were used as secondary antibody and added for 1 h at room temperature. After washing, tetramethyl benzidine solution (Scytec) was added to each well. After a 5-min revelation, the reaction was blocked by adding a stop solution (1 M H_2_SO_4_) and the plates were analyzed at 450 nm with an ELISA reader (Bio-Rad Laboratories). A standard curve for IgG was included in each plate to enable the accurate quantification of anti-αGal antibodies. For the standard curves, wells were coated with serial dilutions of human polyclonal IgG (starting at 2.5 ng per well; ChromPure human IgG, whole molecule). Background plates (that is, plates that were not coated with the αGal antigen) were prepared in parallel to measure nonspecific binding of the sera and background readings were subtracted from the readings obtained in the αGal-coated plate. In addition, a pool of sera from 11 healthy individuals was included in each plate and was used as an interassay internal quality control of the results.

### Measurement of anti-Neu5Gc IgG by EIA

Human sera samples were obtained from the Translink clinical centers and used in accordance with the Declaration of Helsinki and Tel Aviv University Institutional Review Board. Specific overall anti-Neu5Gc IgG reactivity in human sera was evaluated while blinded to patient/control status by an ELISA against coated mouse serum Neu5Gc-containing sialoglycoproteins, as described by Padler-Karavani et al.^64^. Briefly, Costar 96-well plates were coated overnight at 4 °C with 1 µg per well WT pooled mouse sera (lacking mouse anti-human IgG) in coating buffer (50 mM sodium carbonate-bicarbonate buffer, pH 9.5). Wells were blocked for 2 h at room temperature with PBS/ovalbumin (OVA) blocking buffer (PBS, pH 7.3, 1% chicken OVA). During blocking, human serum was diluted 1:100 in EIA buffer (PBS, pH 7.3, 1% chicken OVA and *Cmah*^−/−^ pooled sera that lacked mouse anti-human reactivity, diluted at 1:4,000) and incubated on ice for 2 h. Next, PBS/OVA was removed from the wells and preincubated human serum was added to triplicate wells at 100 µl per well then incubated at room temperature for 2 h. Wells were washed three times with PBS, pH 7.3, 0.05% Tween 20 (PBST) by AquaMax 2000 plate washer (Molecular Devices); detection antibody was then added (100 µl per well, 1:7,000 HRP goat anti-human IgG diluted in PBS) and incubated for 1 h at room temperature. After washing three times with PBST, wells were developed with 140 µl of 0.5 mg/ml *O*-phenylenediamine in 100 mM citrate-PO_4_ buffer, pH 5.5; the reaction was stopped after 20 min with 40 µl H_2_SO_4_ (4 M), then absorbance was measured at an optical density (OD) of 490 nm on a SpectraMax M3 (Molecular Devices).

### Statistical analyses of the Translink cohort human sample studies

Results are expressed as the mean ± s.d. for continuous variables or as count and percentage for categorical variables. Comparisons between groups were performed using Student’s *t*-tests or chi-squared tests for continuous or categorical variables, respectively. Univariate pairwise comparisons were used to assess the evolution of anti-Neu5Gc and anti-αGal IgGs according to time (Kruskal–Wallis test with Dunn/Dunnett/Bonferroni adjustment or followed by two-stage linear step-up procedure of Benjamini, Krieger and Yekutieli to correct for multiple comparisons by controlling the FDR, as recommended by Prism v.8 (GraphPad Software) and described in context).

The first objective of the study was to assess the effect of implanting a BHV on antibody levels, including the anti-Neu5Gc and anti-αGal IgGs. How the markers (anti-Neu5Gc IgG, anti-αGal IgG) changed over time consisted of two phases: a first phase after surgery corresponding to the early inflammatory response due to CPB and/or bioprosthesis implantation; and a second phase to assess the long-term inflammatory evolution. Therefore, we performed a two-phase linear mixed-effect model for each longitudinal marker^65^ with a continuity at the slope changepoint to take into account interindividual and intraindividual variabilities. The changepoint time was determined by the likelihood profile of the models. We used a logarithmic transformation of antibody values to respect both assumptions related to the normality and homoscedasticity of residuals and linear relationship over time. Three random effects were considered for the baseline value and each linear slope. In the multivariate analysis, the effect of BHV implantation was adjusted on any variable that reached a *P* < 0.20 in the univariate analysis. The final model was estimated through a backward procedure performed manually variable by variable with the use of a Wald test (*P* < 0.05). This procedure allows the identification of possible confounding factors (variation of regression coefficients >20%). The linear mixed model assumed a missing at random process for the antibody levels^66^. This assumption seemed reasonable in our context (short follow-up of four years after surgery and only four deaths in the B1 cohort). The second objective of the study was to assess the time between surgery and occurrence of echocardiographic early SVD diagnosis (death censored). A two-tailed *P* ≤ 0.05 was considered statistically significant. Statistical analyses for the clinical study were performed with R v.4.0.3. Other statistical studies were done in Prism v.8 and described depending on the context (for example, nonparametric Mann–Whitney *U*-test; two-way analysis of variance (ANOVA); Bonferroni multiple comparisons test; one-way ANOVA; Dunnett multiple comparisons test).

The association between covariates and antibody levels was tested. The following preoperative data were considered as possible covariates: operative age (years); sex; body mass index (BMI); family history; history of high blood pressure; diabetes mellitus; dyslipidemia; obesity; history of use of tobacco; atrial fibrillation; chronic obstructive pulmonary disease; peripheral vascular disease; renal failure (Cockcroft–Gault creatinine clearance <60 ml min^−1^); left ventricular ejection fraction (<50% and <30%); center name; and implanted prosthesis.

### Tissue samples of BHVs and explanted calcified valves

The commercial bioprostheses used in the study were donated by Medtronic 3f, Sorin SOLO, St Jude Medical, St Jude Epic, St Jude Trifecta and Carpentier–Edwards. Samples were stored at 4 °C until use. Porcine pericardium from WT, *Ggta1*^−/−^ knockout strain (Gal knockout) and double knockout *Ggta1*^−/−^/*Cmah*^−/−^ strain (Gal/Gc double knockout; porcine double knockout) were kindly provided by Avantea. Tissues were collected into protease inhibitor solution at 4 °C, fixed with glutaraldehyde (2.5% glutaraldehyde and 2.5% formaldehyde in 0.1 M PBS) and shipped to Tel Aviv University at 4 °C. Human explanted calcified bioprosthetic or native human valves were collected by surgery, stored in PBS and then immediately shipped to Tel Aviv University at 4 °C.

### Tissue homogenization of BHVs and explanted calcified valves

This was performed as described by Reuven et al.^12^ with some modifications. Briefly, refrigerated (4 °C) human explanted calcified bioprosthetic or native human valves were weighed, then samples (approximately 60 mg) were finely sliced and dissolved in homogenization buffer (1 ml Tris-HCl buffer 50 mM, pH 5.5, 1 mM Ca^2+^, 1 mM PMSF, 2 mg ml^−1^ of type 2 collagenase). The solution was thoroughly vortexed for 30 s then incubated at 37 °C for 1 h while shaking at 220 r.p.m. Samples were then put on ice and sonicated with a probe sonicator (Sonic Dismembrator; Thermo Fisher Scientific) 3 times at medium power, each for 10 s with 30-s interval incubation on ice. Sonicated tissues were inserted into a glass Dounce tissue homogenizer (5 ml; Sigma-Aldrich) and homogenized first with a loose pestle (20 times each for BHV and 10 times each for native valve tissues), then with a tight pestle (20 times each for BHV and 10 times each for native valve tissues). The protein content in the homogenate was evaluated by a standard bicinchoninic acid (BCA) assay according to the manufacturer’s protocol (Pierce). The homogenate was kept at −20 °C until use.

### Detection of Neu5Gc in BHV homogenates by ELISA

Homogenates of commercial BHVs (Medtronic 3f, Sorin SOLO, St Jude Medical, St Jude Epic, St Jude Trifecta, Carpentier–Edwards) were analyzed by ELISA, as described by Reuven et al.^12^. Tissue homogenates were coated onto 96-well plates (Costar) at 2 µg per well in duplicates in 50 mM sodium-bicarbonate buffer, pH 9.5, and incubated overnight at 4 °C. Wells were blocked for 1 h at room temperature with blocking buffer (PBS, pH 7.4, 1% OVA (grade V; Sigma-Aldrich)). Wells were aspirated and incubated with diluted primary antibody (chicken anti-Neu5Gc IgY, 1:1,000 dilution, 10 µg ml^−1^) at 100 µl per well in the same blocking buffer for 2 h at room temperature. The plates were washed 3 times with PBST (PBS, pH 7.4, 0.1% Tween 20) and subsequently incubated for 1 h at room temperature with HRP-conjugated secondary antibody in PBS (HRP donkey anti-chicken IgY, 1:3,000 dilution, 0.26 µg ml^−1^). After washing 3 times with PBST, wells were developed with 140 µl of 0.5 mg ml^−1^ *O*-phenylenediamine in 100 mM citrate-PO_4_ buffer, pH 5.5; the reaction was stopped with 40 µl H_2_SO_4_ (4 M). Absorbance was measured at 490 nm on a SpectraMax M3. Specific binding was defined by subtracting the background readings obtained with the secondary antibody only on coated wells.

### Sialic acid content analysis by DMB–HPLC

The sialic acid content in tissue homogenates was analyzed as described by Reuven et al.^12^. Sialic acid was released from glycoconjugates by acid hydrolysis with 2 M acetic acid for 3 h (neutralizing with 0.1 M NaOH) at 80 °C. Free sialic acid was then derivatized with DMB (Sigma-Aldrich) for 2.5 h at 50 °C, separated by Microcon-10kDa centrifugal filters and analyzed by fluorescence detection on reverse-phase HPLC (Hitachi HPLC Chromaster). HPLC was run on a C18 column (Phenomenex Gemini C18, 250 × 4.6 mm) at 24 °C in running buffer (84.5% double-distilled H_2_O, 8.5% acetonitrile, 7% methanol (Merck)) for 60 min at a flow rate of 0.9 ml min^−1^. Quantification of sialic acid was done by comparison with known quantities of DMB-derivatized Neu5Ac^67^.

### IHC of BHVs

This was performed as described by Paul et al.^68^ with some modifications. Commercial BHV (punches or tissue discs) (Trifecta and Sorin SOLO, bovine pericardial tissues) were fixed in 4% paraformaldehyde, transferred into 30% sucrose solution, then embedded onto optimal cutting temperature (OCT) compound (Scigen). 30-µm sections (MICROM HM 450; Thermo Fisher Scientific) were transferred into a tissue cryopreservation solution (50 mM PBS containing 28.6% ethylene glycol and 23.8% glycerol) in a 96-well plate and stored at −20 °C until staining. For in-solution staining, each section collected with a painting brush was washed for 5 min in PBS in a 48-well plate 3 times, incubated in blocking solution (PBS, 0.1% Tween 20, 5% fish gelatin; Sigma-Aldrich) for 1 h at room temperature, then stained in a 96-well plate with 100 µl of primary antibody in blocking buffer (chicken anti-Neu5Gc IgY or control IgY, 1:2,000 dilution, 5 µg ml^−1^) for 3 h at room temperature. To demonstrate specific Neu5Gc staining, the primary antibody was preincubated in blocking buffer at 4 °C for 1 h with control 20 mM Neu5Ac, pH 7.0, or control Neu5Ac glycoproteins (10% human serum) or inhibiting 20 mM Neu5Gc, pH 7.0, or inhibiting Neu5Gc glycoproteins (10% chimpanzee serum). After 3 h of incubation for staining with pretreated primary antibody, sections were washed in PBS 3 times, 5 min each, then incubated with secondary antibody HRP donkey anti-chicken IgY (1:500 dilution; 1.56 µg ml^−1^) in PBS for 1 h at room temperature, then developed with 3,3-diaminobenzidine reagent (Enco) for 3 min, followed by 3 washes with PBS. Stained sections were mounted in Fluoromount-G (SouthernBiotech) and a coverslip was placed gently over the slide; staining was captured in a Nikon Eclipse Ti-S microscope under a bright field objective. The refractive index of the 10× objective (MRL00042) was 0.30 and images were captured with a Nikon DS-U3 DS-Filc-U3 camera.

### Immunofluorescence of calcified BHVs

This was performed as described by Cohen et al.^69^ with some changes. The refrigerated leaflet tissue of explanted calcified valves was frozen in OCT compound and kept at −80 °C until use. Before sectioning, tissue blocks were transferred to −20 °C for 24 h, then placed in a cryostat chamber (Bright Instruments Limited) to reach −20 °C. Then 20-µm cryosections (Leica DB80 LX microtome blades) were placed onto positively charged slides (adhesion slides; Bar-Naor), air dried for 30–60 min, then stored at −20 °C. Cryosections were thawed then blocked (PBS, 0.1% Tween 20, 1% BSA) for 1 h at room temperature, followed by endogenous biotin blocking (used for biotinylated primary antibodies), washing with PBS three times, then staining with primary antibody in blocking buffer (mouse anti-human C5b-9 IgG, 0.01 mg ml^−1^; Abcam; or biotinylated rabbit anti-human TNF-α IgG, 0.1 μg ml^−1^) overnight at 4 °C in a humid chamber, while sections were covered with parafilm. Slides were washed with PBS three times, then incubated with secondary antibodies (Cy3 goat anti-mouse IgG, 0.0075 mg ml^−1^ or Cy3 streptavidin, 0.005 mg ml^−1^, respectively) at room temperature for 45 min, washed with PBS and nuclei counterstained with 0.1 µg ml^−1^ DAPI in PBS for 5 min, followed by a final PBS wash and mounting in Fluoromount Aqueous Mounting Medium using a coverslip. Slides were imaged with an inverted LSM 510 META confocal microscope (20×/0.4 objective; ZEISS) with bright field and fluorescence channels. Images were analyzed using the LSM Image Browser version 5.

### Purification of human IgG from explanted calcified valves

Refrigerated (4 °C) human explanted calcified bioprosthetic or native human valves were homogenized in PBS, pH 7.4, containing 1 mM PMSF in a Dounce homogenizer (with loose then tight pestle; 20 times each for BHV and 10 times each for native valve tissues), centrifuged at 10,000*g* for 5 min then the supernatant was incubated with protein A Sepharose 4 Fast Flow beads or with protein G Sepharose 4 Fast Flow beads at 4 °C for 48 h while shaking gently. Bound IgG was loaded onto a polyprep column (Bio-Rad Laboratories) at 4 °C, then eluted with 0.1 M citric acid, pH 3.0, and neutralized with 2 M Tris-HCl (pH 8.0). The eluate was concentrated in a 10 K Amicon centrifugal filter at 5,000*g* to 50 µl in PBS, protein-quantified by BCA assay (Thermo Fisher Scientific), then immediately analyzed by glycan microarray at 20 ng µl^−1^. IgG purified from homogenized calcified native/BHV explants by protein A (or protein G) were examined on a glycan microarray at 40 ng µl^−1^ (HHV-1 to HHV-16; BHV-1 to BHV-5) or at 20 ng µl^−1^ (BHV-6 to BHV-8) probed with Cy3 goat anti-human IgG (0.4 µg ml^−1^). To evaluate the IgG subclass in these samples, 96-well Costar plates were coated with IgG purified from explanted BHVs by protein A or protein G (1 µg per well), then the levels of human IgG subclasses were evaluated by ELISA as described above (detection of Neu5Gc in BHV homogenates by ELISA**)**, only that the detection antibodies were HRP-conjugated mouse anti-human IgG1, IgG2, IgG3 or IgG4 diluted 1:300 in PBS, followed by washing, developing and reading of signals from slides.

### Sialoglycan microarray analysis

Microarrays were manufactured with synthetic Neu5Ac and Neu5Gc glycans each containing a linker with a terminal primary amine^18,70^ using NanoPrint LM-60 Microarray Printer (Arrayit) on epoxide-derivatized slides (PolyAn 2D) with 16 subarray blocks on each slide (v.8.1), as described elsewhere^18,70^. Serum samples were diluted 1:100, then analyzed as described previously^12,18,39^. Briefly, slides were rehydrated with distilled H_2_O and incubated for 30 min in a staining dish with 50 °C prewarmed 0.05 ethanolamine in 0.1 M Tris-HCl, pH 9.0, to block the remaining reactive epoxy groups on the slide surface, then washed with 50 °C prewarmed distilled H_2_O. Slides were centrifuged at 200*g* for 3 min, then fitted with ProPlate Multi-Array 16-well slide module (Invitrogen) to divide into the 16 subarrays (blocks). Slides were washed with PBST (PBS, pH 7.4, 0.1% Tween 20), aspirated and blocked with a 200-µl subarray of PBS/OVA blocking buffer (PBS, pH 7.3, 1% chicken OVA) for 1 h at room temperature with gentle shaking. Next, the blocking solution was aspirated and a 100-µl block of primary antibody (human or mouse serum samples diluted 1:100) in PBS/OVA was added, then slides were incubated at room temperature with gentle shaking for 2 h. Slides were washed three times with PBST then with PBS for 5 min per wash with shaking; then binding of primary antibodies (human IgG or mouse serum) were detected with 1.5 μg ml^−1^ Cy3 goat anti-human IgG (H+L) or Cy3-AffiniPure goat anti-mouse IgG diluted in PBS at 200 µl per block, then incubated at room temperature for 1 h. Slides were washed three times with PBST, then with PBS for 5 min per wash followed by removal from the ProPlate Multi-Array slide module. Slides were immediately dipped in a staining dish with distilled H_2_O and were incubated for 10 min with shaking followed by centrifugation at 200*g* for 5 min; then dry slides were scanned immediately.

### Array slide processing

Processed slides were scanned and analyzed at a 10-μm resolution with a Genepix 4000B Microarray Scanner (Molecular Devices) using 350 gain, as described by Leviatan et al.^18^. Images were analyzed with the Genepix Pro 6.0 software (Molecular Devices). Spots were defined as circular features with a variable radius and local background subtraction was performed. Data were analyzed with Microsoft Excel Version 16.54.

### Affinity purification of anti-Neu5Gc antibodies

Antibodies were affinity-purified from pooled human IgG (Privigen IVIg)^71^ or human serum samples, as described previously by Lu et al.^72^, only that sequential columns of serum glycoproteins of *Cmah*^−/−^ mice (Neu5Gc-deficient) and C57BL/6 mice (Neu5Gc^+^) were used. Anti-Neu5Gc antibodies were affinity-purified from three representative sera (V-0168, V-0165, P-0067) at two time points (at inclusion (month 0) and at one month after implantation (month 1). To evaluate the IgG subclass, 96-well Costar plates were coated with these affinity-purified serum anti-Neu5Gc antibodies (1 µg per well), then the levels of human IgG subclasses were evaluated by ELISA as described above and detected by HRP-conjugated mouse anti-human IgG1, IgG2, IgG3 or IgG4 diluted 1:300 in PBS.

### Mice

Neu5Gc-deficient *Cmah*^−/−^ C57BL/6 mice were bred and maintained according to the Animal Care and Use Committee protocol approved by Tel Aviv University. The dark–light cycle was 12 h (7:00–19:00), ambient temperature was kept at 22 ± 1 °C and humidity was maintained at 50%.

### BHV calcification model in naïve *Cmah*^−/−^ mice

Commercial BHVs were examined: Sorin SOLO (two samples), St Jude Medical Trifecta (three samples), Carpentier–Edwards and Medtronic 3f (two samples). Each BHV was cut into seventeen 6-mm diameter discs (KRUUSE biopsy punch); 8 discs were pretreated with affinity-purified human anti-Neu5Gc IgG (10 µg ml^−1^ in PBS) and another 8 discs were treated with control PBS (each disc in 500 µl), incubated for 48 h at 4 °C, then implanted subcutaneously on the back of *Cmah*^−/−^ mice. Four discs were implanted in each mouse (two discs on each side), two mice per treatment (antibody or control pretreated discs). For implantation, 6–7-week-old mice were anesthetized subcutaneously (1:1:8 of ketamine:xylazine:saline; 10 µl per g of weight); then the skin was sectioned and the pretreated discs gently washed in PBS were implanted subcutaneously using sterile forceps, followed by sewing of the cut skin. During surgery special care was taken to place the discs deep inside the subcutaneous compartment away from the suture. Mice were killed after one month and discs were explanted for further analysis.

### Quantification of calcium in explanted discs

Calcium content in explanted BHV discs was quantified by atomic absorption spectrometry. Each disc was frozen separately in a punctured Eppendorf at −80 °C for 2 h, then lyophilized, weighed and transferred into acid‐resistant glass tubes; then 25 µl of 12 N HCl was added. Tubes were incubated in a heat block at exactly 100 °C for 1 h, until the sample was completely dehydrated, then reconstituted with 2.97 ml double-distilled H_2_O followed by the addition of 30 µl lanthanum (final 0.1 N HCl with 0.05% lanthanum oxide). Calcium in the samples was measured by atomic absorption spectrophotometer (PerkinElmer 2380) and quantified against Ca^2+^ standard curves (20–0.125 p.p.m.; mg l^−1^) calculated by mg l^−1^ Ca^2+^ × l × dilution factor/mg tissue.

### Immunization of *Cmah*^−/−^ mice with Neu5Gc glyconanoparticles

Nano-ghost glyconanoparticles were prepared from the porcine red blood cells of two strains: αGal *Ggta1*^−/−^ knockout (Neu5Gc^+^; nano-ghost glyconanoparticles^+^) and control double knockout *Ggta1*^−/−^/*Cmah*^−/−^ (Neu5Gc^−^; nano-ghost glyconanoparticles^−^), as described by Reuven et al.^39^. Protein content was determined by BCA assay, volume was adjusted to 2 mg ml^−1^ in double-distilled H_2_O and 1-ml aliquots were stored at −80 °C until used. For immunization, 6–8-week-old *Cmah*^−/−^ mice were immunized intraperitoneally with either nano-ghost glyconanoparticles^+^ or nano-ghost glyconanoparticles^−^. Nano-ghost glyconanoparticles at a 2 mg ml^−1^ protein concentration were mixed 1:1 (by volume) with Freud’s complete adjuvant (FC) until emulsified, then 200 µl were injected intraperitoneally (200 µg nano-ghost glyconanoparticles per mouse), followed by 3 boost immunizations with Freud’s incomplete adjuvant (FIC) at weeks 1, 2 and 6. To evaluate the developed anti-Neu5Gc antibody response and kinetics, mice were bled (facial vein) before immunization; at week 8, the collecting tubes were incubated at room temperature for 1 h, then centrifuged at 17,000*g* for 2 min and the serum was collected. Serum samples were stored at −80 °C until analyzed by glycan microarrays at 1:100 dilution.

### Preparation of mouse anti-Neu5Gc and control sera

Sera from *Cmah*^−/−^ mice immunized with either Neu5Gc^+^ (nano-ghost glyconanoparticles^+^) or control Neu5Gc^−^ (nano-ghost glyconanoparticles^−^) glyconanoparticles were collected and pooled (mounted immune response confirmed by glycan microarrays). To remove the immune response against the nanoparticle carrier, nano-ghost glyconanoparticles^−^ (2 mg ml^−1^) were mixed 1:1 with sera from mice immunized with nano-ghost glyconanoparticles^+^ or nano-ghost glyconanoparticles^−^ in total 1 ml, incubated at 4 °C while shaking for 30 min, then ultracentrifuged at 37,000*g* for 30 min (acceleration 9, deceleration 2). The supernatant was collected and the procedure repeated 4 more times. Serum preclearance against carrier reactivity was confirmed by ELISA against coated nano-ghost glyconanoparticles^−^ (2 µg per well), with 1:200 precleared mouse serum detected with HRP goat anti-mouse IgG (1:5,000).

### BHV calcification model in immunized *Cmah*^−/−^ mice

Six to eight-week-old *Cmah*^−/−^ mice were immunized intraperitoneally with either nano-ghost glyconanoparticles^+^ or control nano-ghost glyconanoparticles^−^ emulsified with FC followed by boost injection with FIC (weeks 1, 2, 6); then immunization was confirmed by glycan microarrays, as described above. On week 10, 1 d before implantation, mice received 1 more boost injection with FIC. Each of the commercial BHVs (Carpentier–Edwards and St Jude Epic) were cut into seventeen 6-mm diameter discs (KRUUSE biopsy punch), 8 discs were pretreated with mouse anti-Neu5Gc sera (nano-ghost glyconanoparticles^+^) and another 8 discs were treated with control-immunized mouse sera (nano-ghost glyconanoparticles^−^). Each disc was immersed in 500 µl of carrier precleared mouse sera diluted 1:100 in PBS, incubated for 48 h at 4 °C, then implanted subcutaneously on the back of *Cmah*^−/−^ mice for 1 month. Four discs were implanted in each mouse (two discs on each side), two mice per treatment (antibody or control pretreated discs). For implantation on week 10, immunized mice were anesthetized subcutaneously (1:1:8 of ketamine:xylazine:saline; 10 µl per g of weight); then the skin was cut and the pretreated discs were gently washed in PBS and implanted subcutaneously using sterile forceps, then the incision was sutured. Mice were killed after 1 month (week 14 after initial immunization) and discs explanted for further analysis.

### Reporting Summary

Further information on research design is available in the Nature Research Reporting Summary linked to this article.

## Online content

Any methods, additional references, Nature Research reporting summaries, source data, extended data, supplementary information, acknowledgements, peer review information; details of author contributions and competing interests; and statements of data and code availability are available at 10.1038/s41591-022-01682-w.

## Supplementary information

Reporting Summary

Supplementary Data 1 Dataset of Extended Data Fig. 1a.

Supplementary Table 1 Supplementary Tables 1–6.

## Data Availability

All data are included in this published article and Supplementary Information. Requests for detailed information regarding the study data can be submitted to the corresponding authors. Data containing protected health information of the Translink participants may be restricted; therefore, such data requests will be reviewed before release.
