# Supplementary material for: The role of antibody responses against glycans in bioprosthetic heart valve calcification and deterioration
Source: Nat Med. 2022 Feb 17;28(2):283–94. doi: 10.1038/s41591-022-01682-w (PMC8863575; doi:10.1038/s41591-022-01682-w)
Supplement: Supplementary file 1 — Reporting Summary [file 41591_2022_1682_MOESM1_ESM.pdf]

nature research

Corresponding author(s): Vered Padler-Karavani

Last updated by author(s): Dec 29, 2021

Reporting Summary

Nature Research wishes to improve the reproducibility of the work that we publish. This form provides structure for consistency and transparency in reporting. For further information on Nature Research policies, see our Editorial Policies and the Editorial Policy Checklist.

Statistics

For all statistical analyses, confirm that the following items are present in the figure legend, table legend, main text, or Methods section.

n/a	Confirmed	
☐	☒	The exact sample size (*n*) for each experimental group/condition, given as a discrete number and unit of measurement
☐	☒	A statement on whether measurements were taken from distinct samples or whether the same sample was measured repeatedly
☐	☒	The statistical test(s) used AND whether they are one- or two-sided<br>*Only common tests should be described solely by name; describe more complex techniques in the Methods section.*
☐	☒	A description of all covariates tested
☐	☒	A description of any assumptions or corrections, such as tests of normality and adjustment for multiple comparisons
☐	☒	A full description of the statistical parameters including central tendency (e.g. means) or other basic estimates (e.g. regression coefficient) AND variation (e.g. standard deviation) or associated estimates of uncertainty (e.g. confidence intervals)
☐	☒	For null hypothesis testing, the test statistic (e.g. *F*, *t*, *r*) with confidence intervals, effect sizes, degrees of freedom and *P* value noted<br>*Give P values as exact values whenever suitable.*
☒	☐	For Bayesian analysis, information on the choice of priors and Markov chain Monte Carlo settings
☒	☐	For hierarchical and complex designs, identification of the appropriate level for tests and full reporting of outcomes
☒	☐	Estimates of effect sizes (e.g. Cohen's *d*, Pearson's *r*), indicating how they were calculated

Our web collection on statistics for biologists contains articles on many of the points above.

Software and code

Policy information about availability of computer code

Data collection	Software used for data collection: Ennov clinical software suite, Ennov EDC, CSonline, version 7.5.20.1, France.
Data analysis	Statistical analyses for clinical study were performed with R software version 4.0.3. Other statistical studies were done in Prism version 8 and described in context

For manuscripts utilizing custom algorithms or software that are central to the research but not yet described in published literature, software must be made available to editors and reviewers. We strongly encourage code deposition in a community repository (e.g. GitHub). See the Nature Research guidelines for submitting code & software for further information.

Data

Policy information about availability of data

All manuscripts must include a data availability statement. This statement should provide the following information, where applicable:

- Accession codes, unique identifiers, or web links for publicly available datasets
- A list of figures that have associated raw data
- A description of any restrictions on data availability

All data are included in this published article and supplementary information. Requests for detailed information of study data can be submitted to the corresponding authors. Data containing protected health information of Translink participants may be restricted, therefore such data requests will be reviewed before release.

Field-specific reporting

Please select the one below that is the best fit for your research. If you are not sure, read the appropriate sections before making your selection.

☒ Life sciences ☐ Behavioural & social sciences ☐ Ecological, evolutionary & environmental sciences

For a reference copy of the document with all sections, see nature.com/documents/nr-reporting-summary-flat.pdf

Life sciences study design

All studies must disclose on these points even when the disclosure is negative.

Sample size

Regarding the clinical study, there is no data available in the literature in order to scientifically justify the size of patients required for the study. The number of patients was defined according to the maximum enrolment capacities in each participant center. The present analysis focused on 1668 subjects operated in 4 European cardiac surgery centres. 1426 patients operated for single aortic valve replacement with a BHV (Group A = 174 patients, Group B1 = 500 patients, Group B2 = 752 patients) and 242 controls mechanical heart valve replacement or coronary artery bypass grafting (Group B1 = 118 controls and Group B2 = 124 controls) (supplementary Table 3).
Regarding other experiments, no sample size calculation was performed but rather study design was based on availability of samples for analysis, in particular of commercial BHVs and explanted valves for all in vitro and in vivo studies in mice.

Data exclusions

No data was excluded

Replication

Reproducibility of all ELISA and glycan microarray assays were confirmed by more than three independent experiments . All anti-Gal and anti-Neu5Gc quantifications included a unified IgG standard curve in each experimental ELISA plate. Glycan microarrays included QC testing after each print run. We used thousands of samples by these methods, including from other cohorts, and all attempts at replication were successful. All other experiments were done at least twice independently or with multiple biological replicates (based on their availability), with similar results. The tissue staining with anti-TNFa of explanted calcified native human valve was tested only once due to limited sample availability (Extended data Fig. 3g). In addition, IgG subclass determination was examined once on 3 biological replicated (Fig. 3d), however the data was reproduced with other samples of affinity-purified anti-Neu5Gc antibodies from 3 other serum samples (Extended data Fig. 1d).

Randomization

This is a prospective, multicenter, international, non-randomized study. Patient's allocation in the BHV patients and control patients groups was decided according to medical indications. To assess the possible contribution of confounding factors after BHV implantation, Group B1 antibody responses were examined through multivariable statistical models.

Blinding

The investigators were blindded to group allocation during investigation

Reporting for specific materials, systems and methods

We require information from authors about some types of materials, experimental systems and methods used in many studies. Here, indicate whether each material, system or method listed is relevant to your study. If you are not sure if a list item applies to your research, read the appropriate section before selecting a response.

Materials & experimental systems

n/a	Involved in the study	
☐	☒	Antibodies
☒	☐	Eukaryotic cell lines
☒	☐	Palaeontology and archaeology
☐	☒	Animals and other organisms
☐	☒	Human research participants
☐	☒	Clinical data
☒	☐	Dual use research of concern

Methods

n/a	Involved in the study	
☒	☐	ChIP-seq
☒	☐	Flow cytometry
☒	☐	MRI-based neuroimaging

Antibodies

Antibodies used

Biolegend: Affinity-purified polyclonal chicken anti-Neu5Gc IgY (Cat#: 146903, 1 µg/ml or 10 µg/ml), Control IgY (Cat#: 402101, 10 µg/ml). Jackson ImmunoResearch: horseradish peroxidase (HRP)-Affipure donkey anti-chicken IgY (IgG) (H+L) (Cat#: 703-005-155, 1:3000 or 1:500), Cy3-goat-anti-human IgG (H+L) (Cat#: 109-165-003, 1.5 µg/ml), Cy3-AffiniPure-goat-anti-mouse IgG Fcγ Fragment Specific (Cat#: 115-005-008, 7.5 µg/ml), HRP-goat-anti-mouse IgG (Cat#: 115-035-071, 1:5000), HRP F(ab`)2-goat-anti-human IgG Fcγ fragment specific (Cat#: AB_2337596; 1:1000), ChromPure human IgG, whole molecule (Cat#: 009-000-003; 1:1000), Cy3-streptavidin (Cat#: 016-160-084, 5 µg/ml). Invitrogen: HRP-Mouse-anti-Human IgG1 Fc (clone: HP6069, Cat#: A-10648; 1:300), HRP-Mouse-anti-Human IgG2 Fc (clone: HP6014 , Cat#: 05-0520; 1:300), HRP-Mouse-anti-Human IgG3 (Hinge) (clone: HP6047 , Cat#: 05-3620; 1:300). Thermo Fisher Scientific: HRP-Mouse-anti-Human IgG4 Fc (clone: HP6025, Cat#: MA1-34437; 1:300). Biotinylated rabbit-anti-human TNFa antibody (PeproTech, Cat#: 500-P31A, 0.1 µg/ml), mouse monoclonal antibody against human C5b-9 (Abcam; clone: aE11, Cat#: AB66768; 10 µg/ml).

Validation	Statements for the species and application of the primary antibodies used were validated in each manufacturer's website.

Animals and other organisms

Policy information about studies involving animals; ARRIVE guidelines recommended for reporting animal research

Laboratory animals	Neu5Gc-deficient Cmah-/- C57Bl/6 mice used both males and females at age 6-8 weeks old. Neu5Gc-deficient Cmah?/? C57Bl/6 mice were bred and maintained according to Animal Care and Use Committee protocol approved by Tel Aviv University. The dark/light cycle was 12 hrs (7 am – 7 pm), ambient temperature of 22+/-1 C, and humidity maintained at 50%.
Wild animals	No wild animals were used in this study
Field-collected samples	No field-collected samples were used in this study
Ethics oversight	Neu5Gc-deficient Cmah-/- C57Bl/6 mice were bred and maintained according to Animal Care and Use Committee protocol approved by Tel Aviv University

Note that full information on the approval of the study protocol must also be provided in the manuscript.

Human research participants

Policy information about studies involving human research participants

Population characteristics	Table 1. Describes all baseline characteristics of the Translink cohort at inclusion. Group A: SVD patients, Group B1: first time BHV recipient or control MHV/CABG treated patients, Group B2: BHV recipients >=4 years prior to inclusion and controls.
Recruitment	Translink is a European (and Canada), prospective, multicenter, open study (Clinical Trial number: NCT02023970), designed to evaluate the kinetics of the immune response before and after implantation of aortic bioprosthetic heart valves (BHVs) up to ~15 years post-treatment. Patients were enrolled into four groups: (1) Group A: BHV recipients with confirmed structural valve deterioration (SVD); (2) Group B1: de novo BHV recipients enrolled before aortic valve replacement with follow-up after 1, 6, 12 and 24 months; (3) Group B2: patients with implanted BHV performed at least 4 years prior to inclusion, with follow-up after 12 and 24 months; and (4) Control B1/B2 groups of non-BHV implanted patients who received mechanical heart valves or patients that required a coronary artery bypass grafting (Fig.1a and Table 1; Supplementary Table 1 describes inclusion and exclusion criteria).<br>Patients enrolled in group B1 were not all consecutives eligible patients as enrollment depended on their acceptance to participate and the availability of research assistants. Enrollment of B2 patients depended on their ability to come to the research hospital, so it is likely that the most remote or disabled patients declined. In addition, patients of control groups were younger as mechanical heart valves are reserved for younger patients. Finally, enrollment of A group patients depended on local research and clinical organization to propose the study during unplanned hospitalization and before invasive therapeutic intervention.
Ethics oversight	The study protocol was reviewed and approved by the European Commission Framework program 7 (EU-FP-7) Ethics Committee, and the Ethics Committees of the five hospitals participating in sample collection: The Nantes University Hospital, in France; The Bellvitge University Hospital (HUB-ICS) of the Catalan Health Institute in Barcelona, Spain; The University Hospital Vall D'Hebron (HUVH-ICS) of the Catalan Health Institute in Barcelona, Spain; The Azienda Ospedaliera di Padova (AOP) in Padua, Italy; St Boniface Hospital in Winnipeg, Manitoba, Canada. All clinical investigations were conducted according to the principles expressed in the Declaration of Helsinki. All study participants provided written informed consent to participate in the trial. Patients samples were shipped to investigational laboratories for immunological studies and used in accordance with the Helsinki declaration and Institutional Review Board of Tel Aviv University and Institutional Review Board of Padua University Hospital.

Note that full information on the approval of the study protocol must also be provided in the manuscript.

Clinical data

Policy information about clinical studies

All manuscripts should comply with the ICMJE guidelines for publication of clinical research and a completed CONSORT checklist must be included with all submissions.

Clinical trial registration	Translink is a European (and Canada), prospective, multicenter, open study (Clinical Trial number: NCT02023970)
Study protocol	NCT02023970 available at clinicaltrials.gov
Data collection	Enrollment in Translink study and patient follow-up were performed at four major European cardiovascular surgery centers in France (University Hospital of Nantes), Italy (University Hospital of Padova), Spain (University Hospitals of Bellvitge, and of Vall d'Hebron), and in Canada (Manitoba) by cardiologists and cardiac surgeons with long experience in clinical research. In each center, BHV-recipients received any of the most widely used and commercially-available BHVs, mainly made of bovine pericardium (~80%; Corevalve/Mosaic/Hancock II of porcine origin).<br>Supplementary Table 1 describes inclusion and exclusion criteria for patients enrolled in groups A, B1, B2.<br>Group A: 174 patients with SVD diagnosis were prospectively enrolled between September 2013 and December 2017. Patients were considered eligible for the study if they presented SVD defined by echocardiography with a mean trans-valvular gradient ≥30 mmHg and effective orifice area (EOA) ≤1 cm2 worsening over time or intra-prosthetic insufficiency > grade 2/4, and leaflet tissue alteration. Patients were enrolled into the trial by cardiologists at each investigational site.<br>Group B1: 500 patients planned for BHV replacement in five European centers were prospectively enrolled between January 2014

and June 2016. Patients were considered eligible for the study if they received only one surgical or percutaneous aortic BHV, either isolated or associated with other procedures such as coronary artery bypass grafting (n=95), mitral or tricuspid valve repair (n=19), radiofrequency (n=13), and Bentall (n=11). Excluded patients were those who received an immunosuppressive regimen before and/or after BHV implantation, who underwent previous cardiac surgery or previous percutaneous BHV implantation, or who needed more than one BHV implantation. B1 patients control group (non-BHV) was formed by 118 patients planned for mechanical heart valve replacement or coronary artery bypass grafting. Group B1 BHV and control non-BHV patients were enrolled into the trial by cardiac sur¬geons and/or cardiologists at each investigational site.
Group B2: Group B2 patients were enrolled during the same period between January 2014 and June 2016, and included 752 patients who underwent BHV replacement at least 4 years prior to enrollment in each center. Patients were considered eligible for the study if they received BHV replacement alone or associated with other procedures such as coronary artery bypass grafting (n=193), mitral or tricuspid valve repair (n=31), radiofrequency (n=27), and Bentall procedure (n=19). The B2 control patients group included 124 patients who underwent mechanical heart valve implantation or coronary artery bypass grafting at least 4 years prior to enrollment. Group B2 BHV patients and control patients were contacted by phone by a clinical research associate, then enrolled into the trial by cardiologists at each investigational site.

Outcomes

Primary Outcome Measures :

Echocardiography data to assess the structural valve deterioration [Time Frame: 5 years]

The primary endpoint to be analyzed in the study is assessment by echocardiography of structural valve deterioration after implantation of pig valve or bovine pericardium valve or equine pericardium valve.

TRANSLINK project aims primarily at establishing the possible role of recipient immune response against biological prosthetic heart valves as a major cause to mid-long-term structural valve deterioration and clinical dysfunction.

Secondary Outcome Measures :

Process of valve degeneration according to the type of BHV [Time Frame: 5 years]
To study the process of valve degeneration according to the type of BHV (porcine, bovine or equine BHV or type of industrial process) and BHV clinical outcome.

Echocardiography examinations were carried out and analyzed by experienced investigators (C.C., N.P., T.L.T., A.R., A.E. and L.B.) on commercial ultrasound systems (GE Vivid series, Waukesha, WI, USA; or Philipps, Andover, MA) and stored (ImageVault and Echopac software, GE Medical Systems, Horten, Norway) in a centralized CoreLab (Nantes, France). Briefly, the Left Ventricular Outflow Tract (LVOT) diameter was cautiously measured in the parasternal long axis view, the LVOT Velocity Time Integral (VTI) and the aortic VTI were measured in the apical 3- or 5-chambers view with pulsed-wave Doppler and continuous-wave Doppler, respectively. The BHV dimensionless index and Effective Orifice Area (EOA) were calculated. After recent implantation of BHV, early SVD was defined according to current recommendations as an increase in mean gradient >10 mmHg or the worsening of at least one grade of intra-prosthetic aortic regurgitation during follow-up, and abnormal leaflet aspect. Definite SVD was characterized on echocardiography with a mean trans-valvular gradient ≥30 mmHg and effective orifice area (EOA) ≤1 cm2 worsening over time or intra-prosthetic insufficiency > grade 2/4 associated with leaflet tissue alteration.
Finally, a computed tomography (CT) scan evaluation of BHV was carried between 12 and 24 months after BHV implantation for assessing calcium scoring of leaflets. A calcium score >0 was considered abnormal and a sign of early SVD.

Large international and prospective patient's cohort and clinical database with a biocollection [Time Frame: 5 years]
To implement a large international and prospective patient's cohort and clinical database with a biocollection (biobank) of patients receiving an aortic BHV to identify immune biomarkers following aortic valve replacement.

Clinic-biological correlations [Time Frame: 5 years]
To analyse clinic-biological correlations prospectively following BHV.
