# Supplementary material for: The role of antibody responses against glycans in bioprosthetic heart valve calcification and deterioration
Source: Nat Med. 2022 Feb 17;28(2):283–94. doi: 10.1038/s41591-022-01682-w (PMC8863575; doi:10.1038/s41591-022-01682-w)
Supplement: Supplementary file 3 — Supplementary Tables 1–6. [file 41591_2022_1682_MOESM3_ESM.docx]

**Extended data**

**Supplementary Table 1.** Inclusion and exclusion criteria of study participants.

| **Inclusion criteria**  **BHV patients**  Age 18–85 years at the time of surgery (written informed consent received prior to enrollment).  Group B1:  Single aortic valve replacement with a BHV (surgical or percutaneous valve), with or without additional procedures such as percutaneous stenting, bypass surgery, mitral or tricuspid valve repair, radiofrequency or Bentall.  First cardiac surgery.  No immunosuppressive treatment 6 months prior and after enrollment.  No use of allo- or xenogenic-derived material besides aortic valve prosthesis during cardiac surgery.  Patient affiliated to a Health Care Social Security.  Group B2:  Aortic valve replacement with a BHV (surgical or percutaneous valve) conducted more than 4 years prior to enrollment, with or without additional procedures such as percutaneous stenting, bypass surgery, mitral or tricuspid valve repair, radiofrequency or Bentall.  Group A:  BHV recipients with echographic signs of SVD:  Mean trans-valvular gradient ≥ 30 mm Hg,  AND effective orifice area ≤ 1 cm^2^ worsen over time,  OR aortic insufficiency > grade 2/4.  Group control non-BHV patients:  Age >65 years for CABG treatment.  Age 18–85 years for MHV replacement due to aortic stenosis that can be combined with CABG or aortic surgery at the same time.  No immunosuppressive treatment 6 months prior and after enrollment.  No use of other allogenic- or xenogenic-derived material during cardiac surgery (bovine or equine pericardium, additional BHV replacements, etc.) |
| --- |
| **Non-inclusion criteria (group B1 and control group of non-BHV patients)**  Severe renal insufficiency: GFR ≤ 30 ml/min.  Severe dyslipidemia: total cholesterol >350 mg/dl, triglycerides >750 mg/dl.  Ongoing infection.  Active autoimmune diseases.  Immunosuppression treatment.  Previous cardiac surgery.  More than one BHV implantation. |

BHV: bioprosthetic heart valve, MHV: mechanical heart valve, CABG: coronary artery bypass grafting, GFR: glomerular filtration rate

**Supplementary Table 2.** Echocardiographic characteristics of Group B1 patients and controls at inclusion baseline (native aortic valve) and after BHV implantation (M6, M12-M24). At second follow-up, 18 patients had early signs of SVD, that in comparison with the remaining 353 patients without signs of SVD, showed significantly higher peak aortic BHV velocity (mean±SD, Student T-test; 3.4±0.4 m/s vs 2.3±0.5 m/s, P<0.001), and significantly higher mean gradient (mean±SD, Student T-test; 28.1±9.0 mmHg vs 12.5±5.1 mmHg, P<0.0001), while significantly lower EOA (mean±SD, Student T-test; 1.03±0.29 cm² vs 1.89±0.53 cm², P<0.0001), respectively. CT-scan calcium scoring of BHV was recorded once at the end of follow-up in a subgroup of Group B1 patients (ancillary study in one center; 123/500).

| **Group B1** | | | | | | |
| --- | --- | --- | --- | --- | --- | --- |
|  | **Before surgery** | | | **BHV-recipients** | | |
| **Echocardiography** | **Control patients (MHV*) at inclusion** | **BHV patients at inclusion** | **p** | **M6** | **M12-24** | **p** |
| No. of subjects | n=57 | n=500 | – | n=421 | n=371 |  |
| LV EF, % | 58±10 | 60 ± 10 | 0.13 | 62 ± 10 | 63 ± 9 |  |
| Peak AV velocity, m/s | 4.1 ± 1.1 | 4.3 ± 0.9 | 0.20 | 2.27 ± 0.46 | 2.35 ± 0.55 | 0.003 |
| Mean gradient, mmHg | 47 ± 24 | 49 ± 19 | 0.38 | 12.1 ± 4.6 | 13 ± 7 | <0.0001 |
| EOA, cm² | 1.2 ± 0.8 | 0.95 ± 0.67 | 0.02 | 1.91 ± 0.58 | 1.84 ± 0.56 | 0.008 |
| Indexed EOA, cm²/m² | 0.66 ± 0.46 | 0.52 ± 0.36 | 0.02 | 1.04 ± 0.30 | 1.00 ± 0.29 | 0.007 |
| **CT scan calcium scoring, AU (n=123)** | – | – | – | – | 4.8 ± 19.8 |  |

LV EF: left ventricular ejection fraction; AV: aortic valve; EOA: effective orifice area; CT: computed tomography; AU: Agaston units. *In the control group, values were recorded only in patients before their implantation treatment with a mechanical heart valve (MHV) replacement.

**Supplementary Table 3.** Summary of samples available for immunological assays. Most samples were accessible for immunological assays at inclusion (mean±SD; 90.8±8.8%), and less during follow-up (M: month), particularly in Group B2 in which patients were older.

| **Group** | **Donor** | **Sample** | **% Anti-Neu5Gc IgG** | **% Anti-Gal IgG** |
| --- | --- | --- | --- | --- |
| **A** | | | | |
|  | Patients | Inclusion | 97.7 | 83.4 |
|  | n=174 |  |  |  |
| **B1** | | | | |
|  | Patients | Inclusion | 94.6 | 98.2 |
|  | n=500 | 1M | 84.9 | 87.8 |
|  |  | 6M | 82.9 | 82.7 |
|  |  | 12M | 78.9 | 79.3 |
|  |  | 24M | 45.6 | 53.4 |
|  | Controls | Inclusion | 76.3 | 96.6 |
|  | n=118 | 1M | 80.5 | 86.4 |
|  |  | 6M | 71.2 | 73.7 |
|  |  | 12M | 76.3 | 76.3 |
|  |  | 24M | 55.9 | 53.4 |
| **B2** | | | | |
|  | Patients | Inclusion | 98.3 | 99.2 |
|  | n=752 | 12M | 32.1 | 32.2 |
|  |  | 24M | 48.4 | 48.4 |
|  | Controls | Inclusion | 83.1 | 80.8 |
|  | n=124 | 12M | 40.8 | 38.5 |
|  |  | 24M | 17.7 | 16.9 |

**Supplementary Table 4.** Impact of the BHV biomaterial on immunological responses in Group B1 BHV-recipients (n=500). The implanted BHVs were of bovine (n=457; 91.4%), equine (n=20; 4%) and porcine (n=23; 4.6%) origin. Anti-Neu5Gc IgG and anti-Gal IgG were logarithmically transformed, then univariable two-phases linear mixed-effect models were performed.

| **Effect of biomaterial (Bovine, Equine, or Porcine)** | | **Estimate** | **SD** | **p** |
| --- | --- | --- | --- | --- |
| **Variable: Anti-Gal IgG** | | | | |
| Baseline level | | | | |
|  | Bovine tissue | 1.3560 | 0.053 | - |
|  | Equine tissue | 1.3611 | 0.2461 | 0.9963 |
|  | Porcine tissue | 1.6503 | 0.2285 | 0.2167 |
| Linear slope, phase 1, (<45 days) | | | | |
|  | Bovine tissue | 0.0106 | 0.0009 | - |
|  | Equine tissue | 0.0156 | 0.0045 | 0.2442 |
|  | Porcine tissue | 0.0147 | 0.0041 | 0.3290 |
| Linear slope, phase 2, (≥45 days) | | | | |
|  | Bovine tissue | -0.0005 | 0.0001 | - |
|  | Equine tissue | -0.0001 | 0.0003 | 0.2552 |
|  | Porcine tissue | -0.0007 | 0.0003 | 0.3592 |
| **Variable: Anti-Neu5Gc IgG** | | | | |
| Baseline level | | | | |
|  | Bovine tissue | 0.6987 | 0.0412 | - |
|  | Equine tissue | 0.8928 | 0.1923 | 0.3245 |
|  | Porcine tissue | 0.6747 | 0.1828 | 0.8980 |
| Linear slope, phase 1, (<50 days) | | | | |
|  | Bovine tissue | 0.0036 | 0.0011 | - |
|  | Equine tissue | 0.0113 | 0.0057 | 0.1871 |
|  | Porcine tissue | 0.0068 | 0.0051 | 0.5383 |
| Linear slope, phase 2, (≥50 days) | | | | |
|  | Bovine tissue | -0.0005 | 0.0001 | - |
|  | Equine tissue | -0.0013 | 0.0006 | 0.2166 |
|  | Porcine tissue | -0.0006 | 0.0005 | 0.7442 |

**Supplementary Table 5.** Multivariate analysis of immunological responses. In group B1, anti-Neu5Gc IgG and anti-Gal IgG were logarithmically transformed, then multivariate two-phases linear mixed-effect models for adjusted longitudinal evolution of antibody responses were performed (n=118, 500 control and BHV patients, respectively). The effect of BHV was adjusted for age at the time of surgery, gender and sample collecting center at the baseline and in each of the two phases.

| **Effect** | | **Estimate** | **SD** | **p** |
| --- | --- | --- | --- | --- |
| **Variable: Anti-Gal IgG** | | | | |
| Baseline level | | | | |
|  | Control patients | 0.8133 | 0.1538 | 0.2538 |
|  | BHV patients | 0.9528 | 0.1606 |  |
| Linear slope, phase 1, (<45 days) | | | | |
|  | Control patients | 0.0158 | 0.0027 | 0.3568 |
|  | BHV patients | 0.0139 | 0.0028 |  |
| Linear slope, phase 2, (≥45 days) | | | | |
|  | Control patients | -0.0021 | 0.0002 | <0.0001 |
|  | BHV patients | -0.0005 | 0.0002 |  |
| **Variable: Anti-Neu5Gc IgG** | | | | |
| Baseline level | | | | |
|  | Control patients | 0.6022 | 0.0530 | 0.2674 |
|  | BHV patients | 0.7171 | 0.3466 |  |
| Linear slope, phase 1, (<50 days) | | | | |
|  | Control patients | 0.0070 | 0.3993 | 0.5334 |
|  | BHV patients | 0.0088 | 0.0092 |  |
| Linear slope, phase 2, (≥50 days) | | | | |
|  | Control patients | -0.0016 | 0.0537 | 0.1675 |
|  | BHV patients | -0.0020 | 0.0009 |  |

**Supplementary Table 6.** List of glycans printed on glycan microarrays and their characteristics. Sia-linkages (Siaα2–3/6/8 linkages; α3, α6, α8, respectively) or underlying skeleton glycans [Lac (lactose; Galβ4Glc), Gal (galactose), Type I (Galβ3GlcNAcβ), GalNAc (*N*-acetylgalactosamine), LacNAc (*N*-acetyllactosamine; Galβ4GlcNAc), Core 1 (Galβ3GalNAcα), and Type IV (Galβ3GalNAcβ)].

| **Glycan ID** | **Structure** | **Sialic Acid Type** | **Sialic Acid Linkage** | **Skeleton** | **Pairs (P) of Neu5Gc/ Neu5Ac glycans** |
| --- | --- | --- | --- | --- | --- |
| 1 | Neu5,9Ac_2_α3Galβ4GlcNAcβO(CH_2_)_3_NH_2_ | Neu5,9Ac_2_ | α3 | LacNAc | P1-Ac |
| 2 | Neu5Gc9Acα3Galβ4GlcNAcβO(CH_2_)_3_NH_2_ | Neu5Gc9Ac | α3 | LacNAc | P1-Gc |
| 3 | Neu5,9Ac_2_α6Galβ4GlcNAcβO(CH_2_)_3_NH_2_ | Neu5,9Ac_2_ | α6 | LacNAc | P2-Ac |
| 4 | Neu5Gc9Acα6Galβ4GlcNAcβO(CH_2_)_3_NH_2_ | Neu5Gc9Ac | α6 | LacNAc | P2-Gc |
| 5 | Neu5Acα6GalNAcαO(CH_2_)_3_NH_2_ | Neu5Ac | α6 | GalNAc | P3-Ac |
| 6 | Neu5Gcα6GalNAcαO(CH_2_)_3_NH_2_ | Neu5Gc | α6 | GalNAc | P3-Gc |
| 7 | Neu5,9Ac_2_α3Galβ3GlcNAcβO(CH_2_)_3_NH_2_ | Neu5,9Ac_2_ | α3 | Type I | P4-Ac |
| 8 | Neu5Gc9Acα3Galβ3GlcNAcβO(CH_2_)_3_NH_2_ | Neu5Gc9Ac | α3 | Type I | P4-Gc |
| 9 | Neu5,9Ac_2_α3Galβ3GalNAcαO(CH_2_)_3_NH_2_ | Neu5,9Ac_2_ | α3 | Core 1 | P5-Ac |
| 10 | Neu5Gc9Acα3Galβ3GalNAcαO(CH_2_)_3_NH_2_ | Neu5Gc9Ac | α3 | Core 1 | P5-Gc |
| 11 | Neu5Acα3Galβ4GlcNAcβO(CH_2_)_3_NH_2_ | Neu5Ac | α3 | LacNAc | P6-Ac |
| 12 | Neu5Gcα3Galβ4GlcNAcβO(CH_2_)_3_NH_2_ | Neu5Gc | α3 | LacNAc | P6-Gc |
| 13 | Neu5Acα3Galβ3GlcNAcβO(CH_2_)_3_NH_2_ | Neu5Ac | α3 | Type I | P7-Ac |
| 14 | Neu5Gcα3Galβ3GlcNAcβO(CH_2_)_3_NH_2_ | Neu5Gc | α3 | Type I | P7-Gc |
| 15 | Neu5Acα3Galβ3GalNAcαO(CH_2_)_3_NH_2_ | Neu5Ac | α3 | Core 1 | P8-Ac |
| 16 | Neu5Gcα3Galβ3GalNAcαO(CH_2_)_3_NH_2_ | Neu5Gc | α3 | Core 1 | P8-Gc |
| 17 | Neu5Acα6Galβ4GlcNAcβO(CH_2_)_3_NH_2_ | Neu5Ac | α6 | LacNAc | P9-Ac |
| 18 | Neu5Gcα6Galβ4GlcNAcβO(CH_2_)_3_NH_2_ | Neu5Gc | α6 | LacNAc | P9-Gc |
| 19 | Neu5Acα6Galβ4GlcβO(CH_2_)_3_NH_2_ | Neu5Ac | α6 | Lac | P10-Ac |
| 20 | Neu5Gcα6Galβ4GlcβO(CH_2_)_3_NH_2_ | Neu5Gc | α6 | Lac | P10-Gc |
| 21 | Neu5Acα3Galβ4GlcβO(CH_2_)_3_NH_2_ | Neu5Ac | α3 | Lac | P11-Ac |
| 22 | Neu5Gcα3Galβ4GlcβO(CH_2_)_3_NH_2_ | Neu5Gc | α3 | Lac | P11-Gc |
| 23 | Neu5,9Ac_2_α6GalNAcαO(CH_2_)_3_NH_2_ | Neu5,9Ac_2_ | α6 | GalNAc | P12-Ac |
| 24 | Neu5Gc9Acα6GalNAcαO(CH_2_)_3_NH_2_ | Neu5Gc9Ac | α6 | GalNAc | P12-Gc |
| 25 | Neu5Acα3GalβO(CH_2_)_3_NH_2_ | Neu5Ac | α3 | Gal | P13-Ac |
| 26 | Neu5Gcα3GalβO(CH_2_)_3_NH_2_ | Neu5Gc | α3 | Gal | P13-Gc |
| 27 | Neu5Acα6GalβO(CH_2_)_3_NH_2_ | Neu5Ac | α6 | Gal | P14-Ac |
| 28 | Neu5Gcα6GalβO(CH_2_)_3_NH_2_ | Neu5Gc | α6 | Gal | P14-Ac |
| 29 | Neu5,9Ac_2_α3GalβO(CH_2_)_3_NH_2_ | Neu5,9Ac_2_ | α3 | Gal | P15-Gc |
| 30 | Neu5Gc9Acα3GalβO(CH_2_)_3_NH_2_ | Neu5Gc9Ac | α3 | Gal | P15-Ac |
| 31 | Neu5,9Ac_2_α6GalβO(CH_2_)_3_NH_2_ | Neu5,9Ac_2_ | α6 | Gal | P16-Gc |
| 32 | Neu5Gc9Acα6GalβO(CH_2_)_3_NH_2_ | Neu5Gc9Ac | α6 | Gal | P16-Ac |
| 33 | Neu5Acα3Galβ3GalNAcβO(CH_2_)_3_NH_2_ | Neu5Ac | α3 | Type IV | P17-Gc |
| 34 | Neu5Gcα3Galβ3GalNAcβO(CH_2_)_3_NH_2_ | Neu5Gc | α3 | Type IV | P17-Ac |
| 35 | Neu5,9Ac_2_α3Galβ3GalNAcβO(CH_2_)_3_NH_2_ | Neu5,9Ac_2_ | α3 | Type IV | P18-Gc |
| 36 | Neu5Gc9Acα3Galβ3GalNAcβO(CH_2_)_3_NH_2_ | Neu5Gc9Ac | α3 | Type IV | P18-Ac |
| 37 | Neu5,9Ac_2_α6Galβ4GlcβO(CH_2_)_3_NH_2_ | Neu5,9Ac_2_ | α6 | Lac | P19-Gc |
| 38 | Neu5Gc9Ac6Galβ4GlcβO(CH_2_)_3_NH_2_ | Neu5Gc9Ac | α6 | Lac | P19-Ac |
| 39 | Neu5,9Ac_2_α3Galβ4GlcβO(CH_2_)_3_NH_2_ | Neu5,9Ac_2_ | α3 | Lac | P20-Ac |
| 40 | Neu5Gc9Ac3Galβ4GlcβO(CH_2_)_3_NH_2_ | Neu5Gc9Ac | α3 | Lac | P20-Gc |
| 41 | Neu5Acα8Neu5Acα3Galβ4GlcβO(CH_2_)_3_NH_2_ | Neu5Ac-Neu5Ac | α3-α8 | Lac | P25’-Ac-Ac |
| 42 | Neu5Acα8Neu5Acα8Neu5Acα3Galβ4GlcβO(CH_2_)_3_NH_2_ | (Neu5Ac)_3_ | α3-α8 | Lac | P25’-Ac-Ac-Ac |
| 55 | Neu5Acα3Galβ4(Fucα3)GlcNAcβO(CH_2_)_3_NH_2_ | Neu5Ac | α3 | Le^x^ | P21-Ac |
| 56 | Neu5Gcα3Galβ4(Fucα3)GlcNAcβO(CH_2_)_3_NH_2_ | Neu5Gc | α3 | Le^x^ | P21-Gc |
| 57 | Neu5Acα3Galβ4(Fucα3)GlcNAc6SβO(CH_2_)_3_NH_2_ | Neu5Ac | α3 | 6S-Le^x^ | P22-Ac |
| 58 | Neu5Gcα3Galβ4(Fucα3)GlcNAc6SβO(CH_2_)_3_NH_2_ | Neu5Gc | α3 | 6S-Le^x^ | P22-Gc |
| 60 | Neu5Acα3Galβ3GlcNAcβ3Galβ4GlcβO(CH_2_)_3_NH_2_ | Neu5Ac | α3 | LNT | P23-Ac |
| 61 | Neu5Gcα3Galβ3GlcNAcβ3Galβ4GlcβO(CH_2_)_3_NH_2_ | Neu5Gc | α3 | LNT | P23-Gc |
| 62 | Neu5Acα3Galβ4GlcNAc6SβO(CH_2_)_3_NH_2_ | Neu5Ac | α3 | 6S-LacNAc | P24-Ac |
| 63 | Neu5Gcα3Galβ4GlcNAc6SβO(CH_2_)_3_NH_2_ | Neu5Gc | α3 | 6S-LacNAc | P24-Gc |
| 64 | Neu5Acα8Neu5Acα3Galβ4GlcβO(CH_2_)_3_NHCOCH_2_(OCH_2_CH_2_)_6_NH_2_ | Neu5Ac-Neu5Ac | α3-α8 | Lac | P25’’-Ac-Ac |
| 65 | Neu5Acα8Neu5Acα8Neu5Acα3Galβ4GlcβO(CH_2_)_3_NHCOCH_2_(OCH_2_CH_2_)_6_NH_2_ | Neu5Ac-Neu5Ac | α3-α8 | Lac | P25’’-Ac-Ac-Ac |
| 66 | Neu5Acα3(Neu5Acα6)Galβ4GlcβO(CH_2_)_3_NH_2_ | Neu5Ac/ Neu5Gc | α3/α6 | Lac | P26-Ac(Ac) |
| 67 | Neu5Acα6(Neu5Gcα3)Galβ4GlcβO(CH_2_)_3_NH_2_ | Neu5Ac/ Neu5Gc | α6/α3 | Lac | P26-Ac(Gc) |
| 69 | Neu5Gcα8Neu5Acα3Galβ4GlcβO(CH_2_)_3_NH_2_ | Neu5Gc-Neu5Ac | α3-α8 | Lac | P25-Gc-Ac |
| 70 | KDNα8Neu5Acα3Galβ4GlcβO(CH_2_)_2_CH_2_NH_2_ | KDN-Neu5Ac | α3-α8 | Lac | P27-KDN-Ac |
| 71 | Neu5Acα8KDNcα6Galβ4GlcβO(CH_2_)_3_NH_2_ | Neu5Ac- KDN | Α6-α8 | Lac | P28-Ac-KDN |
| 72 | Neu5Acα8Neu5Gcα3Galβ4GlcβO(CH_2_)_3_NH_2_ | Neu5Ac-Neu5Gc | α3-α8 | Lac | P25-Ac-Gc |
| 73 | Neu5Acα8Neu5Gcα6Galβ4GlcβO(CH_2_)_3_NH_2_ | Neu5Ac-Neu5Gc | α6-α8 | Lac | P28-Ac-Gc |
| 74 | KDNα8Neu5Gcα3Galβ4GlcβO(CH_2_)_2_CH_2_NH_2_ | KDN-Neu5Gc | α3-α8 | Lac | P27-KDN-Gc |
| 75 | Neu5Gcα8Neu5Gcα3Galβ4GlcβO(CH_2_)_3_NH_2_ | Neu5Gc-Neu5Gc | α3-α8 | Lac | P25-Gc-Gc |
| 76 | Neu5Acα8Neu5Acα6Galβ4GlcβO(CH_2_)_3_NH_2_ | Neu5Ac-Neu5Ac | α6-α8 | Lac | P28-Ac-Ac |
| 77 | Neu5GcMeα8Neu5Acα3Galβ4GlcβO(CH_2_)_2_CH_2_NH_2_ | Neu5GcMe-Neu5Ac | α3-α8 | Lac | P25-GcMe-Ac |
| 78 | Galα3Galβ4GlcNAcβO(CH_2_)_3_NH_2_ | N/A | N/A | LacNAc | αGal |
